# Computation-accelerated discovery of the K_2_NiF_4_-type oxyhydrides combing density functional theory and machine learning approach

**DOI:** 10.3389/fchem.2022.964953

**Published:** 2022-08-26

**Authors:** Qiang Bai, Yunrui Duan, Jie Lian, Xiaomin Wang

**Affiliations:** College of Materials Science and Engineering, Taiyuan University of Technology, Taiyuan, China

**Keywords:** hydrogen energy materials, oxyhydrides, hydride ion, thermodynamic stability, first principles calculations, machine learning

## Abstract

The emerging K_2_NiF_4_-type oxyhydrides with unique hydride ions (H^−^) and O^2-^ coexisting in the anion sublattice offer superior functionalities for numerous applications. However, the exploration and innovations of the oxyhydrides are challenged by their rarity as a limited number of compounds reported in experiments, owing to the stringent laboratory conditions. Herein, we employed a suite of computations involving ab initio methods, informatics and machine learning to investigate the stability relationship of the K_2_NiF_4_-type oxyhydrides. The comprehensive stability map of the oxyhydrides chemical space was constructed to identify 76 new compounds with good thermodynamic stabilities using the high-throughput computations. Based on the established database, we reveal geometric constraints and electronegativities of cationic elements as significant factors governing the oxyhydrides stabilities via informatics tools. Besides fixed stoichiometry compounds, mixed-cation oxyhydrides can provide promising properties due to the enhancement of compositional tunability. However, the exploration of the mixed compounds is hindered by their huge quantity and the rarity of stable oxyhydrides. Therefore, we propose a two-step machine learning workflow consisting of a simple transfer learning to discover 114 formable oxyhydrides from thousands of unknown mixed compositions. The predicted high H^−^ conductivities of the representative oxyhydrides indicate their suitability as energy conversion materials. Our study provides an insight into the oxyhydrides chemistry which is applicable to other mixed-anion systems, and demonstrates an efficient computational paradigm for other materials design applications, which are challenged by the unavailable and highly unbalanced materials database.

## 1 Introduction

Mixed-anion compounds beyond homoanionic materials impart intriguing properties by the virtual of the anionic diversity in ionic radius, electronegativities and polarizability (Kageyama et al., 2018; Kobayashi et al., 2018; Zapp et al., 2021; Maeda et al., 2022). In particular, oxyhydrides with the coexistence O^2-^ and H^−^ in the anion sublattice offer a superior functionality for materials design, as exemplified in electrolytes (Kobayashi et al., 2016; Takeiri et al., 2019; Matsui et al., 2020; Nawaz et al., 2020; Takeiri et al., 2022), catalysts (Kobayashi et al., 2017) and precursors for topochemical reaction (Masuda et al., 2015; Yajima et al., 2015; Mikita et al., 2016). The lightest mass, large polarizability and high redox potential (-2.3 V) of hydride ions enable the oxyhydrides as novel energy storage and conversion materials (Kobayashi et al., 2016; Liu et al., 2019; Takeiri et al., 2019; Matsui et al., 2020; Nawaz et al., 2020; Lavén et al., 2021; Maeda et al., 2022; Takeiri et al., 2022), and the complex interplay between H^−^ with unique electronic configurations and O^2-^ qualifies the oxyhydrides as magnetic devices (Hayward et al., 2002; Bridges et al., 2005; Yajima et al., 2022). Thanks to the unique characteristics of hydride ions, the discovery of oxyhydrides standing for the frontier of chemistry will open an exciting chemical space serving various applications.

**FIGURE 1:**
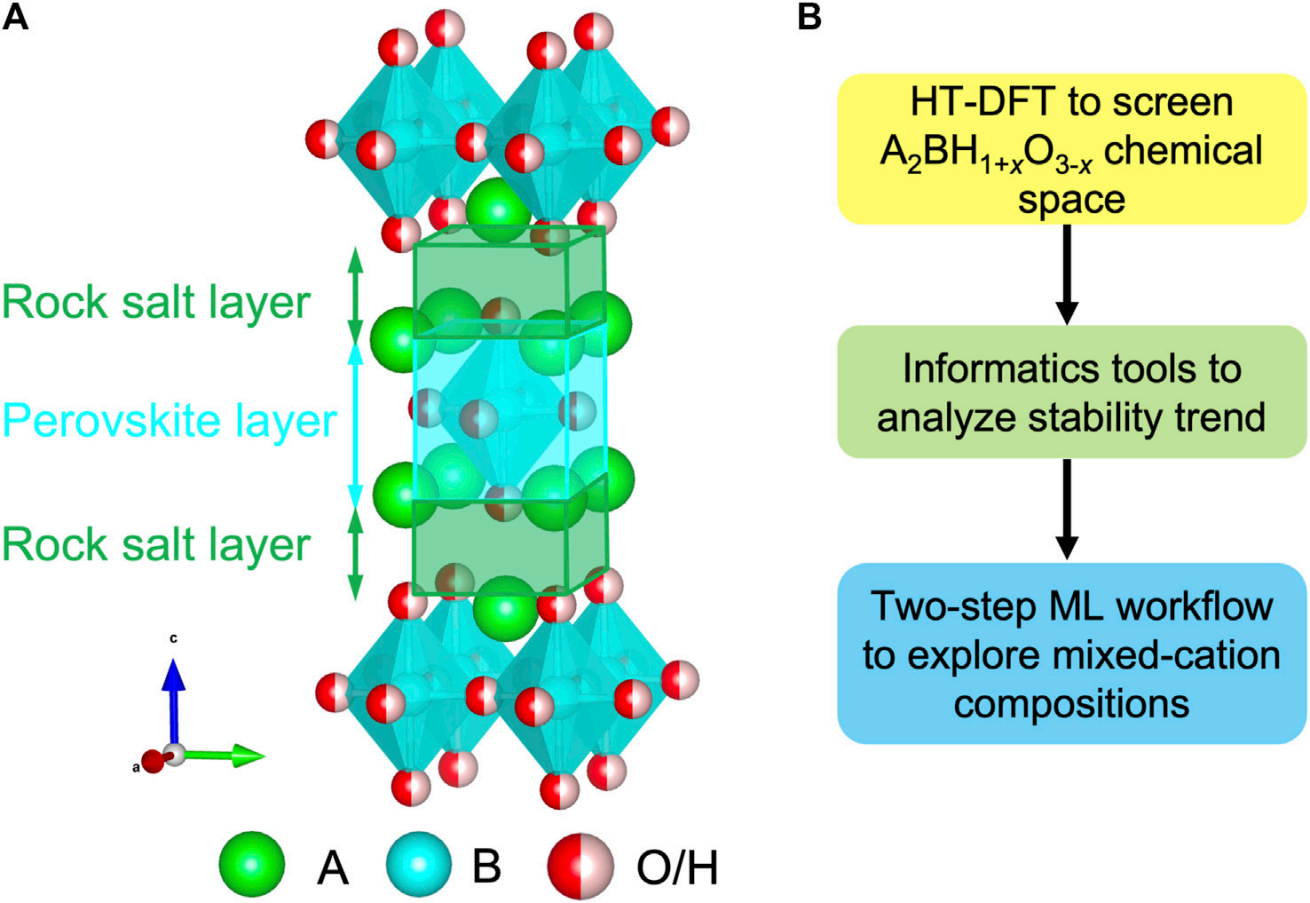
**(A)** Crystal structure of the A_2_BH_1+*x*_O_3-*x*_ (*x* = 0, 1, 2) oxyhydrides in the K_2_NiF_4_-type framework. **(B)** Computational workflow to study the chemistry of the oxyhydrides.

In contrast to massive single-anion compounds, the stable oxyhydrides necessitate robust structural frameworks to accommodate distinctive H^−^ and O^2-^, resulting in the scarcity of the materials (Yamaguchi, 2016; Bai et al., 2018; Kageyama et al., 2018; Kobayashi et al., 2018). Recently, a series of oxyhydrides in the K_2_NiF_4_-type structure with an A_2_BH_1+*x*_O_3-*x*_ (*x* = 0, 1, 2) formula were synthesized in experiments and drew a broad interest with promising properties (Figure 1A) (Kobayashi et al., 2016; Fjellvåg et al., 2019; Takeiri et al., 2019; Matsui et al., 2020; Nawaz et al., 2020; Takeiri et al., 2022). The K_2_NiF_4_-type structure belongs to a member of Ruddlesden-Popper family consisting of a rock salt and perovskite layer stacked along the *c* direction (Figure 1A) (Kobayashi et al., 2018). A breakthrough study successfully utilized La_2_LiHO_3_ as solid-state electrolytes with hydride ions as charge carriers, and demonstrated the exceptionally high H^−^ conductivities in its A-mixed LaSrLiH_2_O_2_ analogs as 0.12 mS/cm at 573 K (Kobayashi et al., 2016). In addition to ionic conductors, Hayward *et al.* discovered La_2_CoH_0.7_O_3_ as magnetic devices arising from its anionic ordering (Hayward et al., 2002; Bridges et al., 2005). Thus, a perovskites-related structural framework provides a tunable sublattice to stabilize the oxyhydrides with excellent functionalities (Bai et al., 2018).

Despite of the rigid framework and attractive properties, the innovations and developments of the K_2_NiF_4_-type oxyhydrides are still in their infancy as only tens of materials (<20) reported in laboratories (Takeiri et al., 2022). The rarity of oxyhydrides is aggravated by the stringent experimental conditions (Tassel et al., 2016; Iwasaki et al., 2018; Kageyama et al., 2018). While the reducing nature of H^−^ demands the strict air/water-free environments (Kageyama et al., 2018), the versatility of anion sublattice to adopt distinctive ions consequently requests the high temperature and pressure conditions (Tassel et al., 2016; Iwasaki et al., 2018). The paucity of the oxyhydrides hinders the progression of applications, and therefore highlights the necessity of expanding their chemistry with high efficiency. High-throughput density functional theory (HT-DFT) computations are demonstrated as efficient tools to explore the uncharted chemical space (Emery et al., 2016; Sun et al., 2019; He et al., 2020; Shen et al., 2021; Wang et al., 2021), helping to guide the laboratory synthesis. In light of the stabilities data generated by the HT-DFT, the stability trend as a function of compound chemistry and their physical origin can be uncovered via the informatics tools (Sun et al., 2019; Ouyang et al., 2021).

Apart from the unmixed crystals (e.g., La_2_LiHO_3_), mixed-cation compounds (e.g., LaSrLiH_2_O_2_) constitute a large portion of compositional space and offer a new dimensionality for targeted properties (Kobayashi et al., 2016; Ye et al., 2018; Matsui et al., 2020). The highest conductivity of a series of the oxyhydrides can be achieved in the A-mixed compounds LaSrLiH_2_O_2_ with 0.21 mS/cm at 590 K (Kobayashi et al., 2016). However, the survey of the mixed materials is challenged by an enormous quantity of their configurations, and therefore calls for more robust search methods such as machine learning (ML) algorithm (Ye et al., 2018; Chenebuah et al., 2021; Talapatra et al., 2021; Tao et al., 2021). While using the ML model to survey materials with promising properties can be extremely powerful, a key roadblock in our task and many similar studies (Cubuk et al., 2019; Jha et al., 2019; Hashimoto et al., 2020; Chen and Ong, 2021; Hanaoka, 2022) to utilize ML approach often points to the unavailability of the database due to their huge quantity. Even though the HT-DFT can be applied to generate the small size database by randomly selecting compounds, the paucity of stable-labeled compounds may disable its usability owing to the highly unbalanced data distribution, which calls for an optimized method that creates the valid database. Since mixed materials share similarity with unmixed compounds in many aspects, it is reasonable to transfer the stability rules learned from the calculated unmixed compounds to preliminarily screen the mixed compounds. Then, the shortlist mixed compounds with high possibility to be stable can be accurately calculated to generate the valid database with a reasonable data distribution for the further ML survey.

In this study, we employed a suite of materials design tools involving HT-DFT, informatics and the ML algorithm to discover and explain the stability relationship of the K_2_NiF_4_-type oxyhydrides (Figure 1B). First, we investigated the stability landscape of the unmixed A_2_BH_1+*x*_O_3-*x*_ oxyhydrides covering 1856 compounds by HT-DFT computations, and identified hundreds of new meta/stable compounds. Then, the genuine materials database inspired us to reveal geometric constraints and electronegativities of cationic elements as significant factors governing the oxyhydrides stabilities via informatics tools. Finally, the mixed-cation oxyhydrides were explored by the two-step ML screening, consisting of a simple transfer learning trained by the unmixed compounds to generate reasonable database and a sequential voting classifier survey. Then, the high hydride conductivities of the selected mixed oxyhydrides indicate their promising applications as energy conversion materials. Our study enhances the understanding of the oxyhydrides chemistry applicable to other mixed-anion systems, and formulates the simple and effective computational workflow for other materials design applications, where a large volume of data is unavailable and the data distribution is highly unbalanced.

## 2 Methods

### 2.1 First principles calculations

All density functional theory (DFT) calculations were performed utilizing the Vienna Ab initio Simulation Package (VASP) (Kresse and Furthmüller, 1996) with the projector augmented-wave (PAW) (Blöchl, 1994) approach and the Perdew–Burke–Ernzerhof (PBE) generalized-gradient approximation (GGA) functional (Perdew et al., 1996). The parameters used in spin-polarized calculations were consistent with the Materials Project (Jain et al., 2011), ensuring the total energies converged to 1 meV per atom. The reciprocal *k-mesh* density was set as 200 per number of atoms to balance the computational cost and accuracy. The valence states of hydrogen in materials were investigated by the Bader (Henkelman et al., 2006; Tang et al., 2009) and Mulliken population analysis implemented in the LOBSTER program (Maintz et al., 2016; Nelson et al., 2020). With reference to the Bader charge of H^−^ in synthesized La_2_LiHO_3_ as -0.64 e^−^ (Bai et al., 2018), we relax the criterion of the hydrogen Bader charge < -0.5 e^−^ to ensure the valence states of H^−^ in all compounds.

Ab initio molecular dynamics (AIMD) simulations were performed in the representative A-mixed oxyhydrides to investigate H^−^ conduction. A Γ-centered 1 × 1 × 1 *k*-point mesh and a time step of 2 fs in NVT ensemble using Nose-Hoover thermostat (Nosé, 1984; Hoover, 1985) were adopted in nonspin-polarized AIMD simulations. Relaxed structures were assigned an initial temperature of 100 K in line with the Boltzmann distribution, and then heated to multiple target temperatures by velocity scaling during 2 ps. The total time of AIMD simulations was at least 50 ps until the diffusivity achieved converged (He et al., 2018). Diffusivities, activation energies and ionic conductivities of hydride ions in the oxyhydrides were obtained based on the established methods (Mo et al., 2011; Bai et al., 2018; He et al., 2018).

### 2.2 High-throughput computations for the oxyhydrides

All calculations for the oxyhydrides were based on supercells with 3 × 3 × 1unit cells and 18 formula units of A_2_BH_1+*x*_O_3-*x*_ (*x* = 0, 1, 2). An orthorhombic unit cell of La_2_LiHO_3_ was used as a prototype structure for A_2_BHO_3_, while a tetragonal unit cell of Sr_2_LiH_3_O served for A_2_BH_2_O_2_ and A_2_BH_3_O compositions following experimental characterizations (Kobayashi et al., 2016). Possible cationic configurations of A_2_BH_1+*x*_O_3-*x*_ were prepared by enumerating elements from the periodic table excluding H, O, S, Se, Te, F, Cl, Br, I, radioactive and noble gas elements. In terms of the common oxidation states for constituting elements (Talapatra et al., 2021), 1856 charge-balanced compounds were selected to be fully optimized via DFT computations. Considering the site selectivity of anions in oxyhydrides (Figure S1 in Supplementary Material) (Kobayashi et al., 2016; Bai et al., 2018), two configurations with the preference of H^−^ over apical and equatorial sites were constructed for each compound, respectively. Thermodynamic stability of the material with the lowest energy was evaluated by the energy above the hull Δ*E*_hull_ (Ong et al., 2008) with respect to competing phases in Materials Project containing 144595 available compounds (Jain et al., 2013). A-mixed A_1_A_2_BH_2_O_2_ compounds with cation mixing were calculated based on the structural model of LaSrLiH_2_O_2_ adopting the La/Sr order with the lowest energies obtained as previous studies (Bai et al., 2018).

### 2.3 Machine learning setup

#### 2.3.1 General work flow

The mixed-cation oxyhydrides were explored by the two-step ML screening. The first ML model was trained and optimized based on the unmixed oxyhydrides to identify compounds with a threshold of Δ*E*_hull_ as 100 meV/atom. Then the trained classification model was transferred to search the A-mixed A_1_A_2_BH_2_O_2_ oxyhydrides, and the mixed compounds predicted to be meta/stable with the possibility >50% were selected and calculated by DFT. The new model was trained and fully optimized based on the newly calculated mixed materials to screen the unstable-labeled materials identified by the initial ML model to retrieve formable compounds for the accurate DFT calculations. The transfer learning in our study refers to transferring the stability rules learned by the unmixed compounds to classify and label the similar A-mixed oxyhydrides, which corresponds to the first round ML training and screening. Since the first- and second-ML models were trained based on the different database, their hyperparameters were fully optimized. All ML computations were performed on the Intel Xeon Platinum 8124M CPU with 18 × 2 cores.

#### 2.3.2 Features generation

The permutation-based importance of 25 features (Table S1 in supplementary material) consisting of 20 elemental properties and five structural/compositional descriptors were evaluated by the random forest algorithm (Breiman, 2001) upon classifying oxyhydrides stabilities. Those empirically considered descriptors are commonly used in other studies to predict unmixed compounds’ stabilities and to analyze stability relationship (Balachandran et al., 2018; Bartel et al., 2019; Chenebuah et al., 2021). The tolerance factor *t*_bv_ (Matsui et al., 2020) that measures the degree of geometric mismatch in the layered-perovskite (K_2_NiF_4_-type) structure is defined as:

$$t_{bv}=\frac{d_{A-X}}{\sqrt{2}d_{B-X}}$$

where *d*_*A-X*_ or *d*_*B-X*_ refers to the bond length between anions X and cation A or B, respectively. The *d*_*A-X*_ and *d*_*B-X*_ were estimated based on the bond valence theory as previous studies (Zhang et al., 2007; Balachandran et al., 2018). The permutation-based feature importance (König et al., 2021) used in calculations can reduce the bias from high cardinality and correlated features, ensuring the accuracy of our analysis.

Due to the complexity of the mixed-cation oxyhydrides, we constructed a dataset of 70 descriptors (Table S2) by applying mathematical operations (e.g., subtract, average, standard deviation) to the original descriptors to identify the stable materials. In order to use the ML model trained by the unmixed compounds to screen the mixed compounds, the feature dimension of the mixed compounds was kept as the unmixed ones. The A-site elemental properties (*f*_A_) were estimated by the arithmetic mean of the two mixed ions (i.e., *f*_A_ = (*f*_A1_ + *f*_A2_)/2) following previous studies (Bartel et al., 2019; Chenebuah et al., 2021; Liu et al., 2022).

#### 2.3.3 Models and features selection

Seven algorithms, i.e., the voting classifier (Kumari et al., 2021) (Note S1), the extra trees classifier (Geurts et al., 2006), the random forest classifier (RFC) (Breiman, 2001), the support vector classifier (SVC) (Shi et al., 2018), the XGBoost (Chen et al., 2015), the gradient boosting classifier (Friedman, 2001) and the decision tree classifier were trained based on the unmixed oxyhydrides to differentiate compounds with a threshold of Δ*E*_hull_ as 100 meV/atom. While multiple metrics (e.g., accuracy and f1 score defined in Note S2) can evaluate the performance of models, f1 score from ten-fold cross-validation was selected as the benchmark for model and features selection, since the number of stable-labeled samples are much fewer (∼5%) than the unstable-labeled samples (∼95%). For each classifier, the sequential forward floating selection algorithm (Note S3) (Ferri et al., 1994; Pudil et al., 1994) was employed to generate a subset of descriptors yielding the highest f1 score from total 70 features and to reduce the overfitting. The classifier with the best f1 score on the validation data and fewer descriptors was applied to predict the formability of A-mixed oxyhydrides following the work flow. The selected features and computation time for each classifier was provided in Table S3-5.

#### 2.3.4 Hyperparameters optimization

The GridSearchCV method implemented in the scikit-learn (Pedregosa et al., 2011) was used to optimize the important hyperparameters of each classifier. The ten-fold cross-validated f1 score were adopted to evaluate the performance of the model during the search. Initially, hyperparameters of each classifier were optimized covering all features for the sequential forward floating selection. After the feature selection, the hyperparameters optimization for each classifier was performed based on the selected features to ensure the better performance. The computation time, parameters search space and their optimized values for each model were in provided in Table S4-7.

## 3 Results

### 3.1 Stability map in the compositional space

**FIGURE 2:**
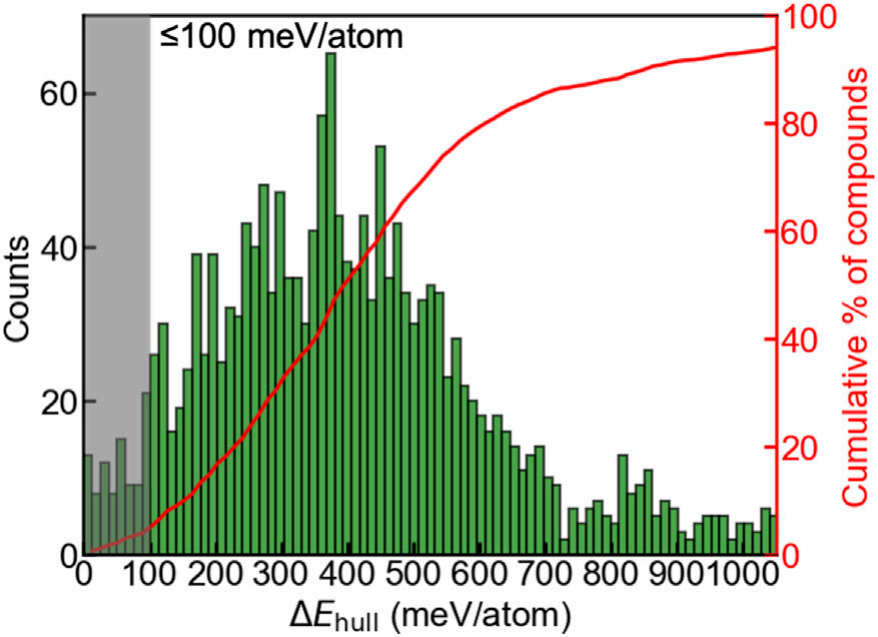
Distribution (left *y*-axis) and the cumulative fraction (right *y*-axis) of the A_2_BH_1+*x*_O_3-*x*_ (*x* = 0, 1, 2) oxyhydrides as a function of Δ*E*_hull_. The shaded area indicates the oxyhydrides with Δ*E*_hull_ ≤ 100 meV/atom and a high likelihood of experimental synthesizability.

We first evaluated phase stabilities of the A_2_BH_1+*x*_O_3-*x*_ (*x* = 0, 1, 2) chemical space by calculating the energy above the hull Δ*E*_hull_ at 0 K. Δ*E*_hull_ of a compound refers to an absolute value of the decomposition energy with reference to competing phases, and serves as an indicator of evaluating the experimental synthesizability, which is widely used in multiple studies (Ong et al., 2008; Sun et al., 2019; Ouyang et al., 2021; Shen et al., 2021; Talapatra et al., 2021; Wang et al., 2021). 80% formable compounds reported from the Inorganic Crystal Structure Database (ICSD) exhibits Δ*E*_hull_ ≤ 100 meV/atom, and the materials with large Δ*E*_hull_ (e.g., ≥100 meV/atom) suffer from the tendency of decomposition and are difficult to synthesize (Sun et al., 2016). Regarding Ba_2_YHO_3_ exhibiting the highest Δ*E*_hull_ as 65 meV/atom in all synthesized oxyhydrides (Table S8) (Nawaz et al., 2020), we relax this criterion and consider a oxyhydride with Δ*E*_hull_ ≤ 100 meV/atom exhibiting a high likelihood of experimental synthesizability. We observe a gaussian-like distribution (Figure 2) of the oxyhydrides as a function of Δ*E*_hull_, with a median Δ*E*_hull_ as 395 meV per atom and a small portion of compounds at the left and right extreme. Only 4.5% oxyhydrides exhibit Δ*E*_hull_ less than 100 meV/atom (Figure 2) awaiting the future experimental realization. The shape of the oxyhydrides distribution with Δ*E*_hull_ is similar to those of oxynitrides and oxyfluorides reported in other studies (Wang et al., 2021), suggesting a similar formation trend in mixed anion systems. The paucity of synthesizable compounds confirmed by our study highlights the necessity of efficient HT computations for materials discovery.

**FIGURE 3:**
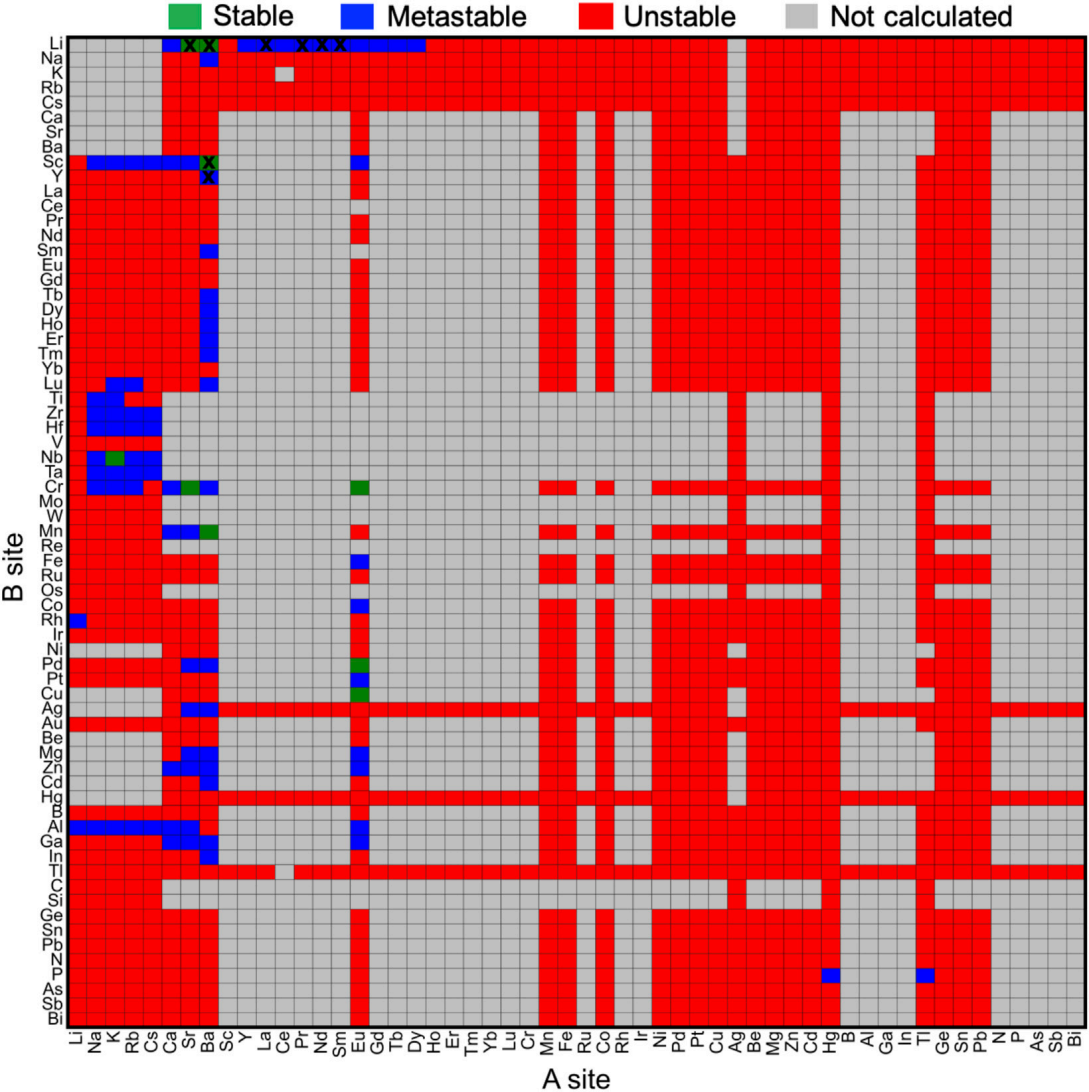
Stability map of the A_2_BH_1+*x*_O_3-*x*_ (*x* = 0, 1, 2) oxyhydrides with elements occupying A and B sites, respectively. Stable (Δ*E*_hull_ = 0 meV/atom) and metastable (0 < Δ*E*_hull_ ≤ 100 meV/atom) oxyhydrides are shown in green and blue, respectively, while unstable oxyhydrides with Δ*E*_hull_ larger than 100 meV/atom are plotted in red. Compounds in gray are not calculated mostly due to the lack of charge-balanced compositions. The blocks marked by black crosses indicate synthesized compounds in experiments. Elements are ordered by the Mendeleev number.

The effect of chemical compositions on the oxyhydrides stability was investigated by constructing stability map (Figure 3) in terms of elements at A and B sites, respectively. While 67 considered elements for the A_2_BH_1+*x*_O_3-*x*_ composition can yield ∼4400 configurations, many compounds fail to satisfy the charge balance criterion (gray blocks in Figure 3) due to the high oxidation states of constituent elements. For example, the elements (e.g., rare earth, B, Al, Ga, In, N, P, As, Sb, Bi, Y and Sc) with +3 oxidation state at A sites can hardly form charge-neutral compositions in the A_2_BH_1+*x*_O_3-*x*_ formula (Figure 3) except for +1 cations (e.g., alkali metals and Ag) at B sites. We excluded those charge-unbalanced compounds from further computations owing to their inherently Coulombic instabilities.

As for other 1856 charge-balanced oxyhydrides, 7 and 77 compounds are found to be thermodynamically stable (Δ*E*_hull_ = 0 meV/atom) and metastable (0 < Δ*E*_hull_ ≤ 100 meV/atom), respectively (Table 1 and S8). The valence states of H^−^ in those newly predicted compounds are confirmed by the Bader analysis (Table S8). All synthesized compounds, i.e., Ln_2_LiHO_3_ (Ln = La, Pr, Nd, Sm) (Kobayashi et al., 2016; Iwasaki et al., 2018), Ba_2_MHO_3_ (M = Y and Sc) (Takeiri et al., 2019; Nawaz et al., 2020) and M_2_LiH_3_O (M = Sr and Ba) (Kobayashi et al., 2016; Takeiri et al., 2022), were identified as meta/stable materials by our compositional screening (Figure 3 and Table 1), confirming the validity of our computations. We note ∼60% synthesized oxyhydrides exhibiting metastability with Δ*E*_hull_ less than 100 meV/atom (Figure 3; Table 1 and Table S8), demonstrating the accessibility of the metastable compounds in laboratory and the essentiality of exploring those materials. Apart from eight synthesized compounds, 76 oxyhydrides are predicted to be meta/stable with a high likelihood of experimental synthesizability, greatly enlarging a library of the oxyhydrides.

**TABLE 1:** Statistics of the known and newly predicted oxyhydrides in terms of Δ*E*_hull_ and compositions. There are eight experimentally synthesized compounds, which are Ln_2_LiHO_3_ (Ln = La, Pr, Nd, Sm) (Kobayashi et al., 2016; Iwasaki et al., 2018), Ba_2_MHO_3_ (M = Sc, Y) (Takeiri et al., 2019; Nawaz et al., 2020) and M_2_LiH_3_O (M = Sr, Ba) (Kobayashi et al., 2016; Takeiri et al., 2022).

Oxyhydrides category	Previously known	Newly predicted	Total number	Percentage
Stable (Δ*E*_hull_ = 0)	3	4^a^	7	0.4% (7/1856)
Metastable (0<Δ*E*_hull_≤100)	5	72^a^	77	4.1% (77/1856)
Unstable (100<Δ*E*_hull_)	0	1763	1763	95% (1763/1856)
Distribution of the oxyhydrides with Δ*E*_hull_≤100				
A_2_BHO_3_	6	37	43	4.6% (43/942)
A_2_BH_2_H_2_	0	21	21	4.2% (21/505)
A_2_BH_3_O	2	18	20	4.9% (20/409)

a Thermodynamically stable and metastable oxyhydrides are screened based on the Bader charge of H less than -0.5, and nine compounds are filtered out.

**FIGURE 4:**
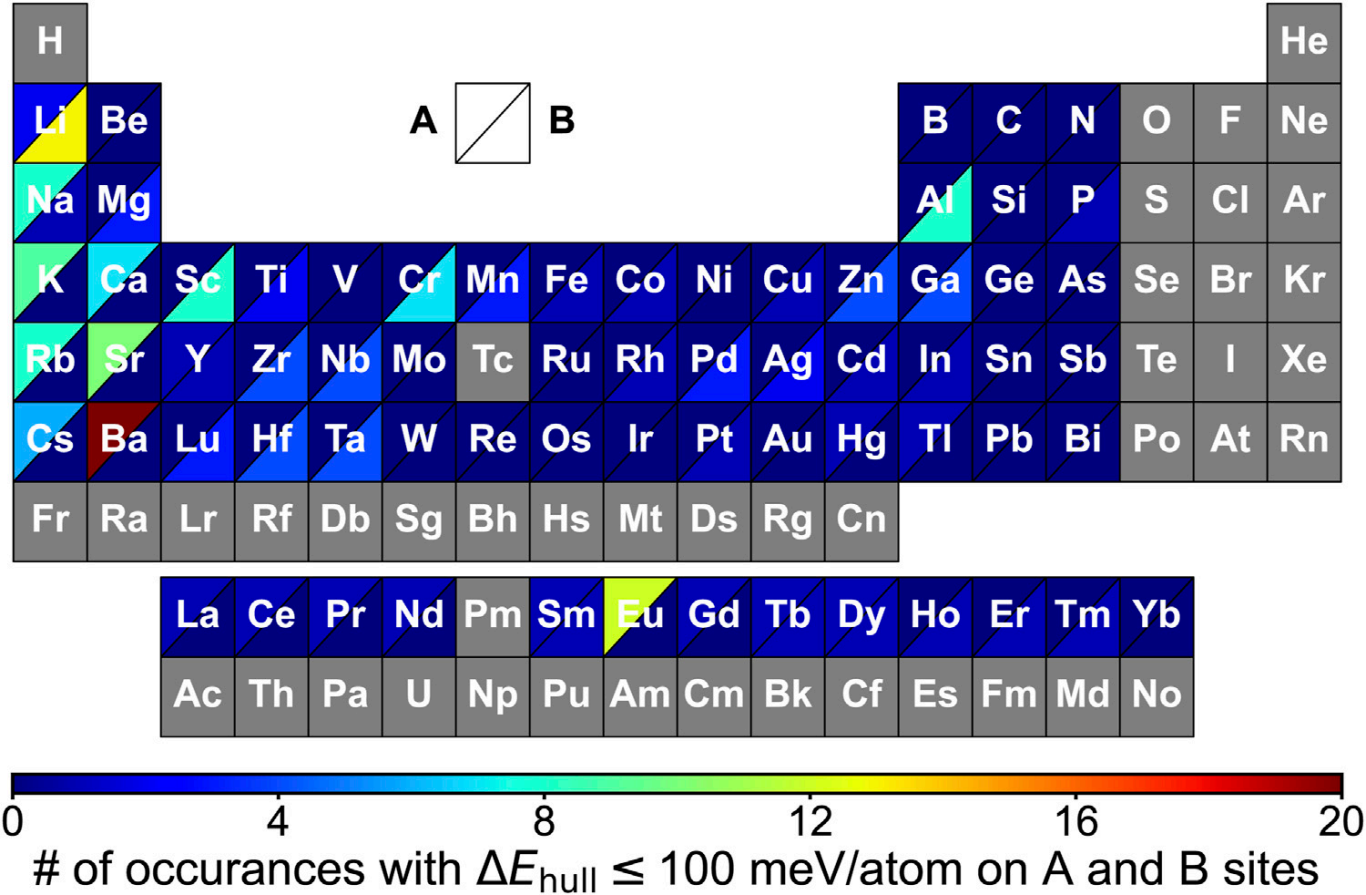
Periodic table showing the frequency of elements in A_2_BH_1+*x*_O_3-*x*_ (*x* = 0, 1, 2) oxyhydrides with Δ*E*_hull_ ≤ 100 meV/atom occupying A and B sites. Each block represents an element that is colored based on the occurrences on A (upper left triangle) and B sites (lower right triangle) in the meta/stable oxyhydrides, respectively. Elements in gray blocks are not considered in the calculations.

The preference of elements occupying A and B sites was visualized by constructing the periodic table in terms of the element’s occurrence in meta/stable A_2_BH_1+*x*_O_3-*x*_ (Figure 4). We observe a high frequency of alkali and alkaline earth metals on the A site with transition metals and p-block elements (e.g., Mg, Al and Ga) on the B site (Figures 3, 4 and Table S8), such as Na_2_AlH_3_O, K_2_ScH_3_O, Sr_2_MgH_2_O_2_ and Na_2_NbHO_3_. Such pattern of the constituting elements in the oxyhydrides in the K_2_NiF_4_ framework is similar to that of perovskites reported by multiple experimental and computational studies due to their structural similarity (Figure 1A) (Emery et al., 2016; Talapatra et al., 2021; Tao et al., 2021).

We note the preference of Li over B sites with lanthanides at A sites in meta/stable compounds (Figures 3, 4), which is consistent with multiple Ln_2_LiHO_3_ (Ln = La, Pr, Nd, Sm) oxyhydrides reported in experiments (Kobayashi et al., 2016; Takeiri et al., 2022). However, other alkali metals except Li cannot form meta/stable oxyhydrides with lanthanides. The physical origin of the distinctive behaviors in alkali metals to form the stable oxyhydrides will be illustrated in Section 3.2. In addition to the explored oxyhydrides, various meta/stable compounds containing novel cations (e.g., Cr, Al, Ga and Mn) at B site are recognized by our computations (Figures 3, 4), with most of those unexplored in experiments. The HT-DFT calculations expand the chemistry of K_2_NiF_4_-type oxyhydrides by providing formable materials with new compositions, and allow us to obtain a complete stability picture in the uncharted chemical space.

### 3.2 Stability trends in the oxyhydrides

**FIGURE 5:**
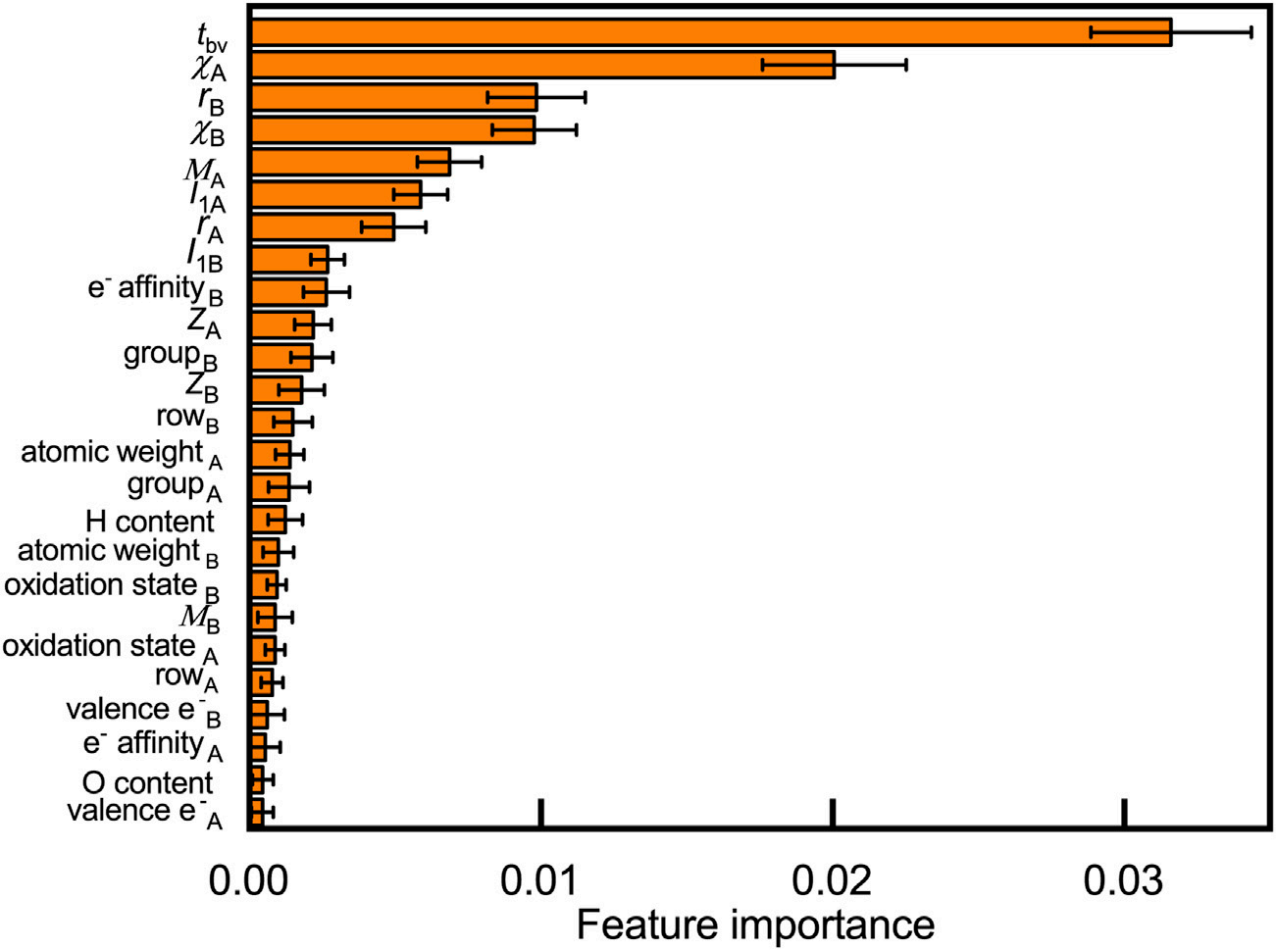
Feature importance to classify oxyhydrides stability based on the permutation method using the random forest model, where 25 features, including 20 basic elemental properties (e.g., Shannon ionic radius *r*, Pauling electronegativities *χ*, Mendeleev number *M*, atomic number *Z*, electrons valence e^−^: the number of valence electrons and *I*_1_: the first ionization energy) and five structural/compositional features (e.g., tolerance factor *t*_bv_, H and O content in composition) are considered. Subscripts of symbols denote corresponding properties of elements at A and B sites, respectively. Errors bars indicate the standard deviation upon shuffling features.

Understanding why certain elements can form stable compounds is a fundamental question that explores the oxyhydrides. We rationalize crucial factors governing materials’ formability by quantifying their contribution to the oxyhydrides stabilities through the random forest (Figure 5) and decision tree analysis (Figure S2). Twenty-five features, including 20 basic elemental properties (e.g., Shannon ionic radius *r*, Pauling electronegativities *χ*, Mendeleev number *M*, atomic number *Z*, and electrons valence e^−^: the number of valence electrons) and five structural/compositional features (e.g., tolerance factor *t*_bv_, H and O content in composition), are considered as possible factors that influence structural stabilities. Those considered descriptors are commonly used in other studies to predict compounds’ stabilities and to analyze stability relationship (Balachandran et al., 2018; Bartel et al., 2019; Chenebuah et al., 2021). We present the contribution of those variables to oxyhydrides stabilities by ranking their feature importance in classifying the oxyhydrides with Δ*E*_hull_ = 100 meV/atom as a threshold (Figure 5, Figure S2). Geometrical descriptors (i.e., *t*_bv_ and *r*_B_) and electronegativities of constituting elements are identified as the most important features differentiating the oxyhydrides. The physical insight relating those features to the oxyhydrides stabilities will be explored in the next subsection.

#### 3.2.1 Geometric factors

**FIGURE 6:**
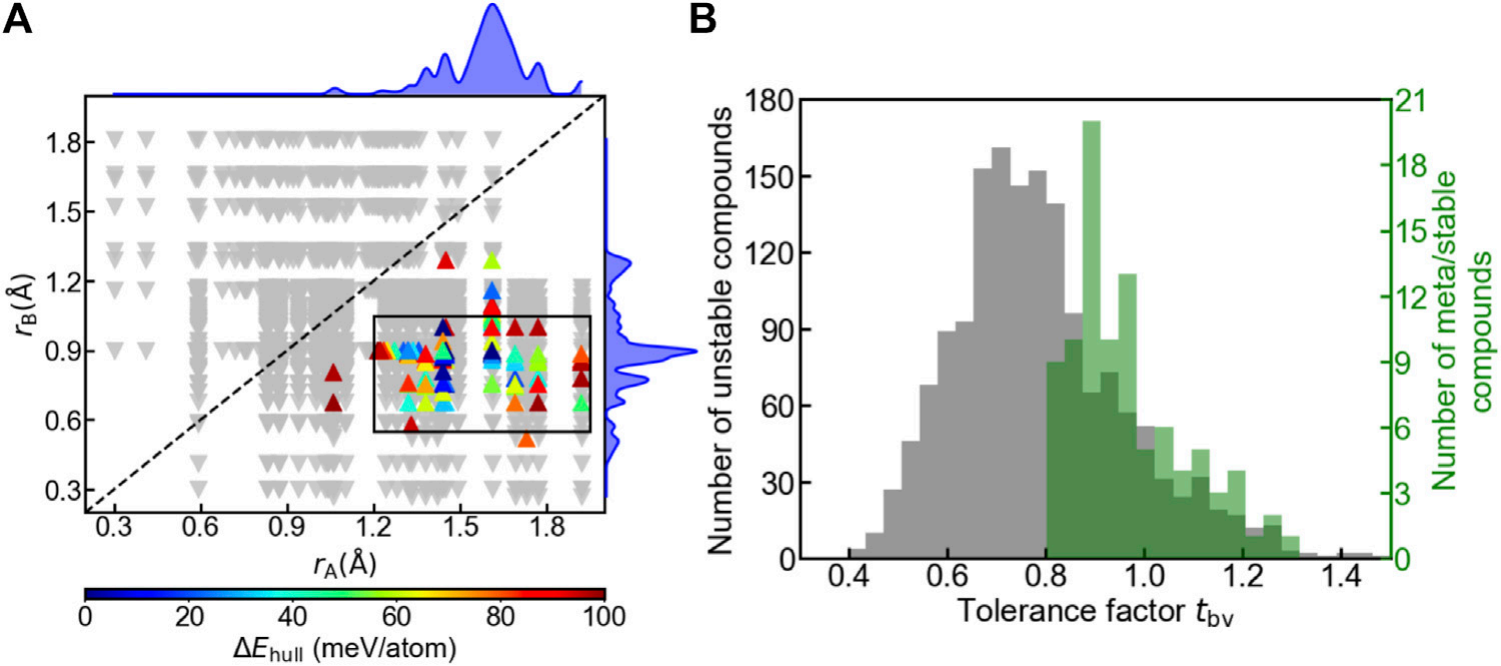
**(A)** Structure map of A_2_BH_1+*x*_O_3-*x*_ (*x* = 0, 1, 2) oxyhydrides with the Shannon ionic radius of ion at A site (*r*_A_) vs that at B site (*r*_B_). Up-pointing triangles indicate meta/stable oxyhydrides (Δ*E*_hull_ ≤ 100 meV/atom), which are colored as a function of Δ*E*_hull_. Those meta/stable compounds are clustered within the rectangular box (0.55 Å < *r*_B_ < 1.05 Å, 1.20 Å < *r*_A_ < 1.95 Å). Down-pointing triangles indicate unstable oxyhydrides (Δ*E*_hull_ > 100 meV/atom). The upper and right curves represent the distribution of meta/stable oxyhydrides as a function of *r*_A_ and *r*_B_, respectively. **(B)** Distribution of the oxyhydrides as a function of the tolerance factor *t*_bv_, where the left and right *y*-axis refer to the number of unstable and meta/stable oxyhydrides, respectively.

We examined the influence of geometry on the oxyhydrides stability by plotting structure map of A_2_BH_1+*x*_O_3-*x*_ (*x* = 0, 1, 2) oxyhydrides with *r*_A_ vs *r*_B_ (Figure 6A). While some stable perovskites still have smaller ions at A sites (Emery et al., 2016), all meta/stable oxyhydrides appear in the lower-right region with *r*_A_ > *r*_B_ (Figure 6A), and demonstrate a tendency of clustering within the rectangular box (0.55 Å < *r*_B_ < 1.05 Å, 1.20 Å < *r*_A_ < 1.95 Å), implicating the strictly geometric requirement to form the stable K_2_NiF_4_ structures. The preference of ionic radius results from the difference between the coordination number of A and B sites and the corresponding space to accommodate the cations, where A-site cations are coordinated by nine anions in comparison with six anions surrounding B sites (Figure 1A). We also utilize the decision tree algorithm to analyze the influence of *r*_A_ and *r*_B_ on the oxyhydrides stabilities (Figure S3). Most stable oxyhydrides (74 out of 93) belong to the node with *r*_B_ < 1.005 Å and *r*_A_ > 1.314 Å (Figure S3). The decision tree analysis indicates stable oxyhydrides tend to have *r*_A_ > *r*_B_, which is consistent with our analysis based on the structure map (Figure 6A).

In addition to ionic radius, tolerance factors *t*_bv_ of the oxyhydrides were investigated, in reference to *t*_bv_ of an ideal K_2_NiF_4_ structure as 1. Most oxyhydrides tend to be meta/stable within the range of 0.8 ≤ *t*_bv_ ≤ 1.2 (Figure 6B), which is consistent with the experimental observation (Matsui et al., 2020). The decision tree analysis also demonstrates most stable oxyhydrides (85 out of 93) are within the range of 0.838 ≤ *t*_bv_ ≤ 1.109 (Figure S4), which is consistent with our analysis based on the *t*_bv_ distribution plot (Figure 6B). This can explain the absence of the stable oxyhydrides with larger alkali metal cations (except Li) occupying B sites and smaller lanthanides at A sites mentioned in Section 3.1 (Figures 3, 4), since their *t*_bv_ are out of range. While there is a high degree of clustering of meta/stable compounds with respect to ionic radius and *t*_bv_, many compounds satisfying those criterions are still unstable (Figure 6), indicating the insufficiency of geometric factors to describe the oxyhydrides stabilities.

#### 3.2.2 Electronegativities

**FIGURE 7:**
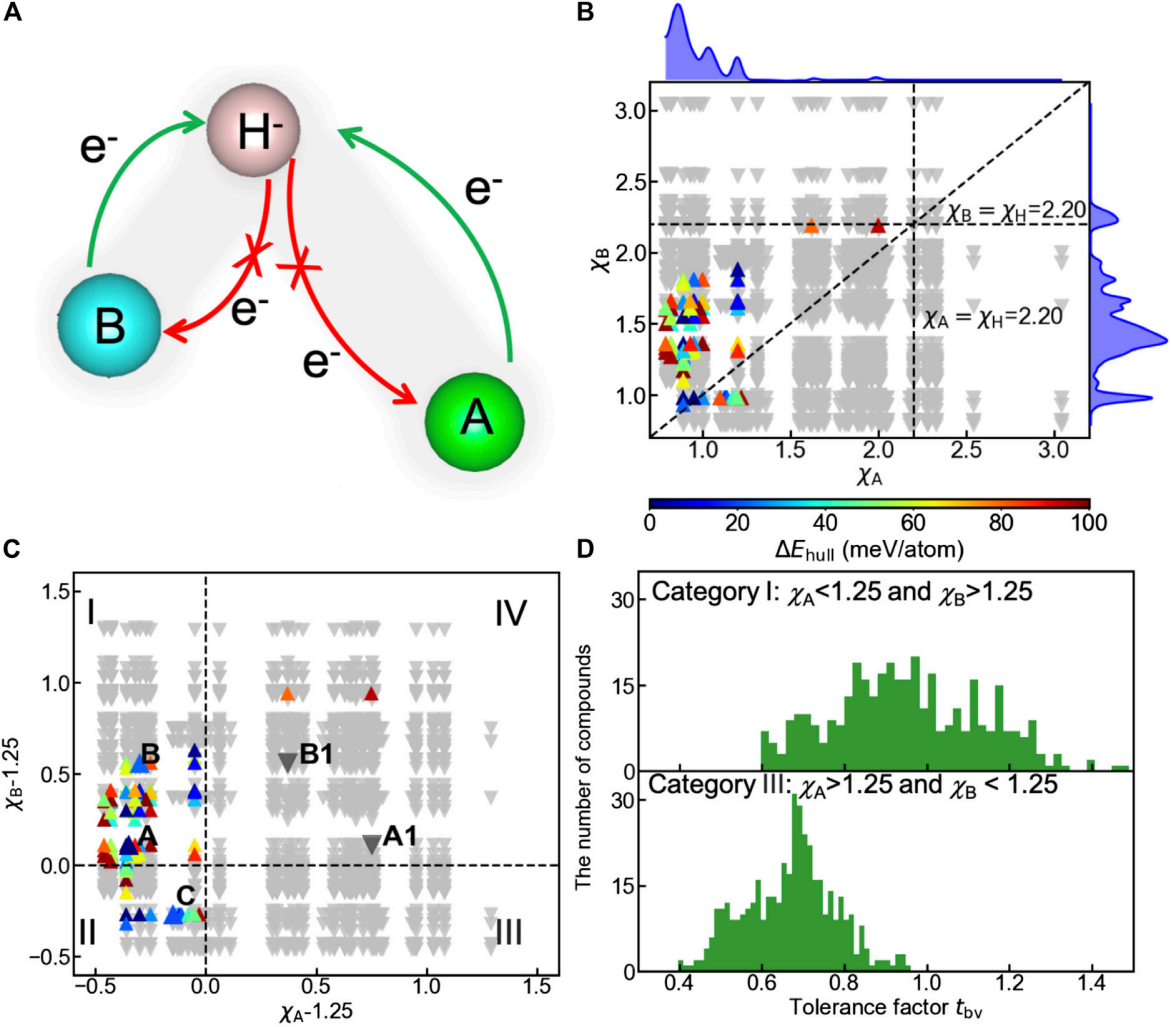
**(A)** Schematic of the charge transfer in hydride bonding to stabilize the oxyhydrides, with bond length of A-H longer than that of B-H. **(B)** Structure map of the oxyhydrides exhibiting the electronegativity of an element at A site (*χ*_A_) vs that at B site (*χ*_B_). The upper and right curves represent the distribution of meta/stable oxyhydrides as a function of *χ*_A_ and *χ*_B_, respectively. **(C)** Structure map of the oxyhydrides with *χ*_A_-1.25 vs *χ*_B_-1.25. The structure map is categorized into four parts (I, II, III and IV) with respect to the threshold of *χ* = 1.25. Five representative oxyhydrides are highlighted, i.e., (A) Ba_2_ScHO_3_ (Δ*E*_hull_ = 0 meV/atom), A1: Hg_2_ScHO_3_ (Δ*E*_hull_ = 366 meV/atom), (B) Sr_2_GaHO_3_ (Δ*E*_hull_ = 22 meV/atom), B1: Tl_2_GaH_3_O (Δ*E*_hull_ = 199 meV/atom) and (C) La_2_LiHO_3_ (Δ*E*_hull_ = 20 meV/atom). Up-pointing triangles indicate stable and metastable oxyhydrides (Δ*E*_hull_ ≤ 100 meV/atom), which are colored as a function of Δ*E*_hull_. Down-pointing triangles indicate unstable oxyhydrides (Δ*E*_hull_ > 100 meV/atom). **(D)** Distribution of *t*_bv_ for the oxyhydrides belonging to category I and III in Figure 7C, respectively.

Apart from geometric constraints, the electronic origin of the oxyhydrides stability can be rationalized by analyzing the charge transfer between cations and hydride ions. To ensure the negative charge of hydride ions, electron density should be donated by cations to hydrogen, as schematically illustrated in Figure 7A. The opposite direction of charge transfer causes the oxidation of hydrogen, and destabilizes the compounds (Figure 7A). Since electronegativities of elements are crucial factors influencing the charge distribution in chemical bonding, we plotted the structure map of the oxyhydrides (Figure 7B) as a function of electronegativity of an element at A site (*χ*_A_) vs that at B site (*χ*_B_). In all meta/stable oxyhydrides (Δ*E*_hull_ ≤ 100 meV/atom), electronegativities of cationic elements at A/B sites are smaller than the electronegativity of H as 2.20 (Figure 7B). Smaller electronegativities of A/B-site elements can prevent the charge transferring from hydrogen to cations and the consequent hydrogen oxidation, contributing to stabilizing the oxyhydrides.

In addition to the inhibition of hydrogen oxidation as a requisite to stabilize the materials, there should be extremely electropositive cations contributing electrons to hydrogen. A large electronegativity between the cationic element and H can facilitate the charge transfer. We find 98% meta/stable oxyhydrides possess at least one extremely electropositive element with *χ* < 1.25 (Figure 7C) to mainly supply electron density. To better visualize the stability trend as a function of electronegativities, compounds are classified into four categories with respective to *χ*_Α_/*χ*_Β_ − 1.25, and 82 out of 84 meta/stable oxyhydrides fall into the category I and II (Figure 7C). Meta/stable compounds belonging to category I with *χ*_Α_ < 1.25 and *χ*_Β_ > 1.25 have A-site cations mainly acting as electron donors to ensure the valence states of hydride ions and to stabilize materials. Those materials (e.g., Ba_2_ScHO_3_ and Sr_2_GaHO_3_) correspond to the high frequency of alkali and alkaline earth metals on the A site with transition metals and p-block elements on the B site as observed in the periodic table (Figure 4). Compounds in category II benefit from two potential electron donors with small *χ* < 1.25, which correspond to multiple Ln_2_LiHO_3_ (Ln = La, Pr, Nd and Sm) oxyhydrides already explored in experiments (Figure 3 and La_2_LiHO_3_ in Table 2). Although the materials in category III possess B-site cations with *χ* < 1.25, the lack of formable oxyhydrides in this group originates from the geometric perspective. *t*_bv_ of compounds in this category exhibit the obvious deviation from the optimal range as 0.8–1.2 (Figure 7D), because the electropositive B-site cations always have large ionic radius to distort the layer-perovskite structures. Few meta/stable oxyhydrides are found in category IV due to the absence of electropositive cations. The exception of Tl_2_PHO_3_ and Hg_2_PH_3_O as metastable compounds in this category might result from the lack of competing phases in the materials database, which underestimates their decomposition energies.

**TABLE 2:** *χ*_A_, *χ*_B_, *t*_bv_, Δ*E*_hull_, Bader and Mulliken charge of hydrogen in Ba_2_ScHO_3_ (A), Hg_2_ScHO_3_ (A1), Sr_2_GaHO_3_ (B), Tl_2_GaH_3_O (B1) and La_2_LiHO_3_ (C).

Symbol	Composition	*χ*_Α_	*χ*_Β_	*t*_bv_	Δ*E*_hull_ (meV/atom)	Bader charge of H (e^−^)	Mulliken charge of H (e^−^)
A	Ba_2_ScHO_3_	0.89	1.36	0.97	0	−0.71	−0.75
A1	Hg_2_ScHO_3_	2.00	1.36	0.84	366	−0.15	−0.39
B	Sr_2_GaHO_3_	0.95	1.81	0.96	22	−0.54	−0.46
B1	Tl_2_GaH_3_O	1.62	1.81	1.05	199	−0.41	−0.23
C	La_2_LiHO_3_	1.10	0.98	0.87	20	−0.64	−0.73

Two groups of representative materials (i.e., A: Ba_2_ScHO_3_, A1: Hg_2_ScHO_3_ and B: Sr_2_GaHO_3_, B1: Tl_2_GaH_3_O) are highlighted to illustrate the strong correlation among electronegativity, charge transfer and phase stability (Figure 7C and Table 2). In contrast to Ba_2_ScHO_3_ (A) and Sr_2_GaHO_3_ (B), the absence of extreme electropositive cations in Hg_2_ScHO_3_ (A1) and Tl_2_GaH_3_O (B1) impedes the charge transfer to anions, which can be manifested by the decreased negative charge of hydrogen from the Bader charge and Mulliken population analysis (Table 2). The failure of the electronic stabilization leads to the increase of Δ*E*_hull_ for those materials (Table 2). Such trend can also be confirmed by the decision tree analysis (Figure S5), where stable oxyhydrides (81 out of 93) belongs to the node with *χ*_B_ < 1.82 and *χ*_A_ < 1.225. Thus, the electronic stabilization of the oxyhydrides demands the constituting elements with *χ* < *χ*_H_ and at least one extremely electropositive cation.

By applying the aforementioned geometric and electronegativity constraints, 228 compounds were selected from 1856 hypothetical compositions, with 74 of those candidates predicted to be meta/stable. The high ratio (32%) of formable oxyhydrides in shortlisted compounds in comparison with 5% by the random guess demonstrates the geometric constraints and elemental electronegativity as significant factors affecting the oxyhydrides stabilities.

### 3.3 Extension to A-mixed oxyhydrides

Besides fixed stoichiometry compounds, mixed-cation oxyhydrides are promising materials, as they provide an enhancement of compositional tunability for targeted properties. For example, A-mixed LaSrLiH_2_O_2_ demonstrated the highest H^−^ conductivity as 0.21 mS/cm at 590 K in experiments (Kobayashi et al., 2016; Matsui et al., 2020). However, an enormous number of possible configurations cause mixed oxyhydrides relatively untapped, placing computational materials discovery as an efficient tool. Taking the A_1_A_2_BH_2_O_2_ oxyhydrides as an example, the quantity of 17618 charge-neutral compounds disables the enumerated DFT calculations due to great expanse, calling for a more robust survey tool. An obvious tendency of the materials stability with respect to elemental and structural features (Section 3.2) reminds us of employing the ML algorithm to differentiate stable mixed-cation compounds.

**FIGURE 8:**
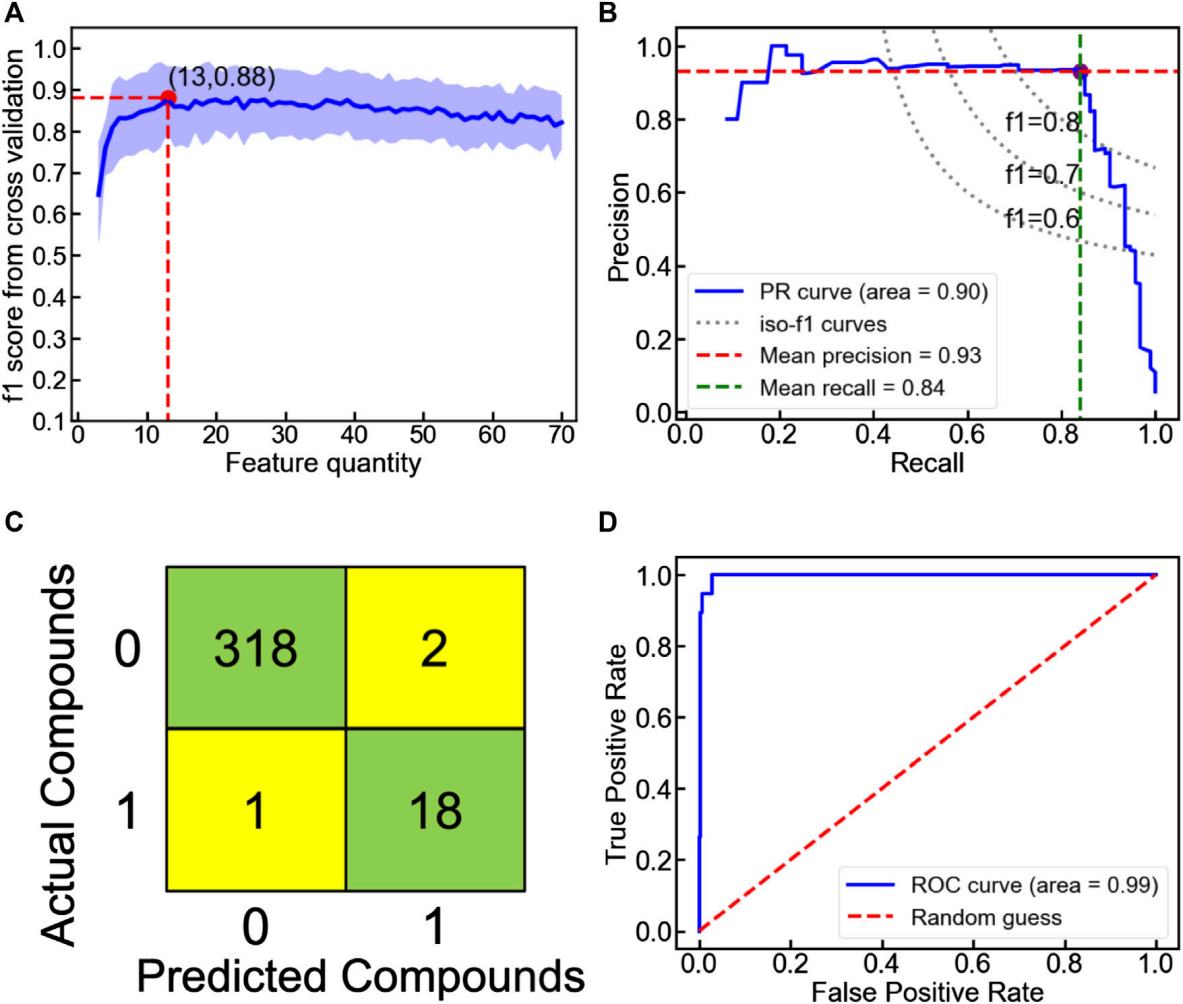
**(A)** Ten-fold cross-validated f1 score as a function of feature quantity in the voting classification model to categorize the unmixed oxyhydrides. The shaded region represents the standard deviation of f1 score upon cross validation. **(B)** Precision-recall, **(C)** confusion matrix and **(D)** receiver operating characteristic (ROC) curves of the voting classification model to categorize the unmixed oxyhydrides upon the ten-fold cross validation.

Since LaSrLiH_2_O_2_ of the A_1_A_2_BH_2_O_2_ composition exhibits the best conduction properties, we choose the A_1_A_2_BH_2_O_2_ oxyhydrides as a model to illustrate our workflow handling the enormous compositional possibilities. Initially, seven candidate classifiers were trained based on the unmixed oxyhydrides dataset and 70 descriptors (Table S2) to differentiate meta/stable compounds (Δ*E*_hull_ ≤ 100 meV/atom). While their classification performance can be evaluated by multiple metrics (e.g., accuracy, f1 score and precision), the highly imbalanced database containing fewer meta/stable oxyhydrides imposes f1 score as a major index for model assessment (Note S2). Among all classifiers, the voting classifier demonstrates the highest cross-validated f1 score as 0.88 with fewer descriptors utilized (Figure 8A; Figure S6; Table 3 and Table S3). Its excellent performance is also proved by the high precision (0.93) and recall rate (0.84) in the precision-recall curve (Figure 8B), with good accuracy (0.99) and separability indicated by the confusion matrix (Figure 8C) and the ROC curve (Figure 8D). Outstanding metrics of the voting model to identify meta/stable compounds indicate its capability of capturing overarching factors impacting oxyhydrides stabilities.

**TABLE 3:** Best f1 scores and the corresponding number of selected features using distinct classifiers models based on the cross-validated data.

Classifier	Best f1 score	Feature quantity
Voting	0.88 (±0.08)	13
Extra trees	0.85 (±0.08)	45
Random forest	0.84 (±0.07)	36
SVC	0.86 (±0.09)	12
XGBoost	0.85 (±0.05)	41
Gradient boosting	0.87 (±0.08)	31
Decision tree	0.77 (±0.08)	69

**FIGURE 9:**
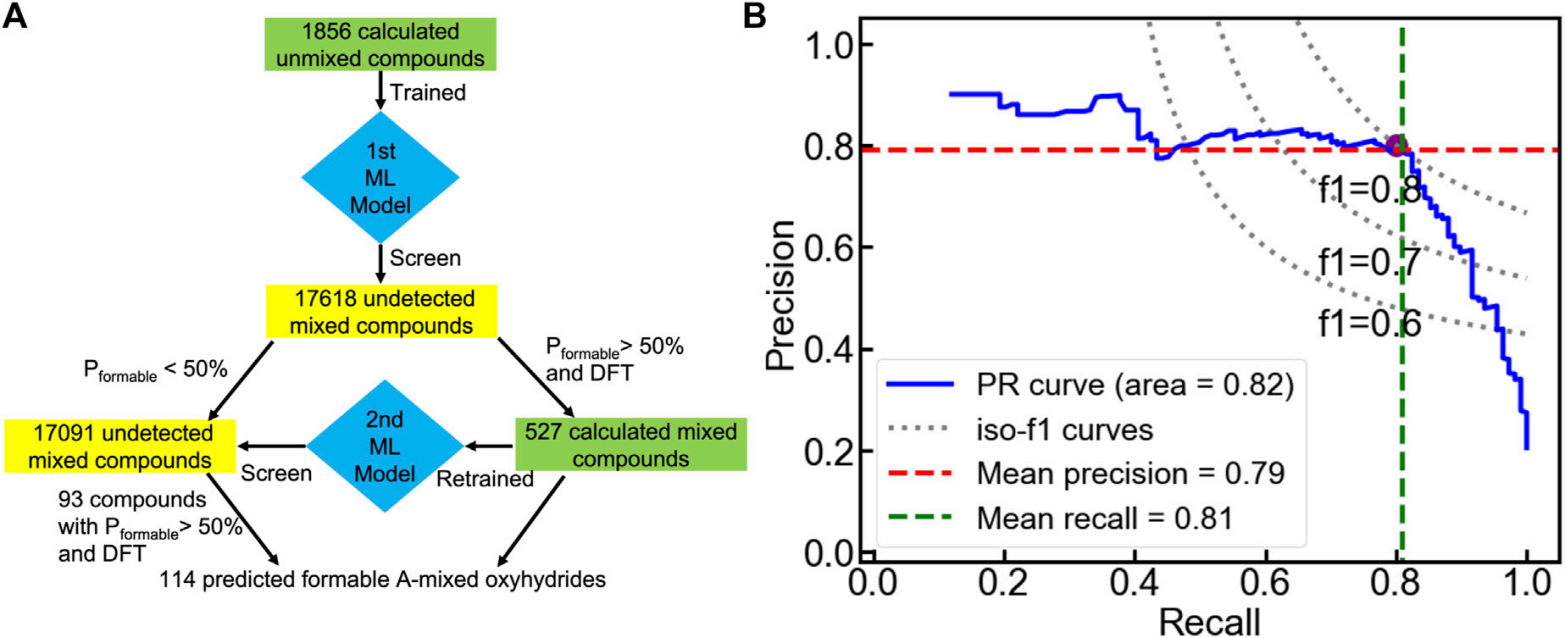
**(A)** Workflow of the sequential machine leaning approach to search formable A-mixed oxyhydrides. **(B)** Cross-validated precision-recall curve of the voting classifier trained from the calculated mixed compounds.

We then transfer the trained classification model to search A-mixed A_1_A_2_BH_2_O_2_ oxyhydrides with good formability (Figure 9A). Among all 17618 electrically neutral configurations, the voting classifier predicts 527 compounds to be meta/stable with a probability larger than 50%. We further performed DFT calculations on those 527 shortlisted materials to determine their thermodynamic stabilities, yielding 110 formable compounds with Δ*E*_hull_ ≤ 100 meV/atom. The simple transfer learning not only accelerates the discovery of stable mixed oxyhydrides, but provides the valid database of the A-mixed oxyhydrides with a reasonable number of stable-labeled compounds for the further ML. Then, a new voting classifier was trained based on those calculated mixed ones to learn the stability rules of the A-mixed oxyhydrides. The retrained model demonstrates the ten-fold cross-validated accuracy and f1 score to identify stable mixed materials as 0.91 ± 0.04 and 0.80 ± 0.09, respectively, demonstrating its excellent ability to identify stable A-mixed crystals (Figure 9B). The new model was used to screen the unstable-labeled materials identified by the initial ML model to retrieve possibly formable compounds for further DFT calculations.

After the two-round ML and DFT screening, 114 A-mixed oxyhydrides are finally found to have Δ*E*_hull_ ≤ 100 meV/atom with a high likelihood of synthesizability (Figure 9A and Table S9). All experimentally synthesized materials (i.e., LnSrLiH_2_O_2_, Ln = La, Nd, Pr, Sm, Gd) (Kobayashi et al., 2016; Matsui et al., 2020) were recognized as formable materials, validating our computation scheme. In addition, the occurrence of new cations (e.g., Zr^4+^, Ce^4+^ at A sites) in the A-mixed oxyhydrides highlights the compositional tunability in mixed compounds (Table S9).

Since ionic conductivity is a key parameter for energy storage materials, we performed AIMD simulations in five randomly selected A-mixed compounds to study their H^−^ diffusivities (Table 4 and Figure S7). Four out of five compounds were predicted to exhibit the facile H^−^ diffusion with σ_Η−_ larger than 0.1 mS/cm at 573 K, except for the σ_Η−_ of KCeMgH_2_O_2_ merely smaller as 0.07 mS/cm. High H^−^ conductivities in those materials demonstrate the potential of the K_2_NiF_4_-type oxyhydrides for a wide range of energy conversion applications, e.g., hydride conductors and fuel cells. Since a library of promising oxyhydrides explored by our data-driven tools constitutes a valuable resource for further discovery, we look forward to a comprehensive investigation on properties of the oxyhydrides to explore their multifunctionality.

**TABLE 4:** Hydride conduction properties of the newly predicted A_1_A_2_BH_2_O_2_ oxyhydrides.

Composition	Δ*E*_hull_ (meV/atom)	*E*_a_ (eV)	σ_Η−_ at 573 K (mS/cm)
CaYLiH_2_O_2_	22	0.24	72
NaLaZnH_2_O_2_	75	0.22	195
NaPrZnH_2_O_2_	78	0.32	76
CaPrLiH_2_O_2_	0	0.56	0.82
KCeMgH_2_O_2_	46	0.67	0.07

## 4 Discussion

Despite of the emerging functionality, the stringent experimental condition causes the oxyhydrides relatively undiscovered, with up to 20 compounds realized in laboratory. Using HT-DFT computations and ML algorithms, we constructed a large stability map of A_2_BH_1+*x*_O_3-*x*_ oxyhydrides. Besides all synthesized compounds identified by our calculations, 185 new compositions with good intrinsic thermodynamic stabilities were discovered to expand the chemical space of the oxyhydrides by ten times. Our prediction of novel oxyhydrides provides a guidance for experimental synthesis, which serves as a first step awaiting future researches that detail their properties.

Based on the established database, underlying features dominating the oxyhydrides stabilities and their physical origin were revealed via informatic tools. Since the meta/stable K_2_NiF_4_-type oxyhydrides exhibit a similar range of *t*_bv_ as 0.8–1.2 with that in perovskite oxides (Li et al., 2013; Emery et al., 2016), we speculate the similar constraint of *t*_bv_ in other stable mixed-anion systems sharing perovskite-related frameworks, such as oxynitrides and oxyfluorides (e.g., ABN_*x*_O_3-*x*_ and A_2_BF_1+*x*_O_3-*x*_) (Li et al., 2013). The criterion of tolerance factor from our analysis can serve as a screening threshold to promote the discovery of other mixed-anion materials from geometric aspect.

In addition to the structural distortion, the bonding between cations and anions plays a crucial role in stabilizing mixed-anion materials. While the upper limit of electronegativities for cationic elements is determined by the anion with a smaller electronegativity in the mixed-anion compounds, the extremely reducing nature of H^−^ further demands a highly electropositive cation to contribute electron density, such as alkali and alkaline earth metals. The trend of cationic electronegativity to stabilize materials may remain valid in other mixed-anion systems due to the similar bonding environment. Taking oxynitrides and oxyfluorides as an example, their requirements of cations in electropositive aspect is more tolerant than that of the oxyhydrides due to their electronegative anions (i.e., N^3-^ and F^−^). Such trend is proved by many compounds (e.g., PbReNO_2_, AgCuF_2_O, TlBiF_2_O) (Wang et al., 2021) containing less electropositive ions realized in experiments. Thus, the rarity of the A_2_BH_1+*x*_O_3-*x*_ oxyhydrides can be attributed to the stringent constraint of cations with far small electronegativity ascribed to the reducing nature of hydride ions.

In order to evaluate the necessity of using the complex voting classifier, we also utilized the widely-used random forest method to search the stable A-mixed oxyhydrides following the two-step ML workflow (Table 5, Table S10 and Figure S8). While the training time of random forest is shorter than of the voting classifier, the hitting rate of the random forest (109/917) is smaller than that of the voting classifier (114/620), leading to calculating additional 300 compounds. The higher precision of the voting classifier may originate from its better performance (e.g., higher f1 score) to identify stable compounds than that of the random forest (Table 3, 5 and Figure S9). Given the computation time of a A-mixed compound by DFT as ∼ 5 hours, calculating 300 more compounds would consume thousands of hours, which is far longer than the difference between the two models’ training time. Thus, it is reasonable to use the voting classifier with the best performance in all classifiers for screening with an acceptable cost. In order to use the ML model trained by the unmixed compounds to screen the mixed compounds, we keep the feature dimension of the mixed compounds as same as the unmixed ones by designating the A-site properties as the arithmetic averages of the mixed ions. Such approximation of the properties for the mixed components is widely used in multiple ML studies (Bartel et al., 2019; Chenebuah et al., 2021; Liu et al., 2022). While the ML algorithm is challenged to learn the complicated effect of the cation mixing on materials’ stabilities (e.g., site miscibility) by averaging site properties (Ouyang et al., 2021) and including those effects by further feature engineering may improve its performance (Talapatra et al., 2021), the ML model still shortlists candidate compounds containing all known materials from thousands of possible configurations, and increases the success rate of finding the formable A-mixed oxyhydrides from ∼0.5% (114/17618) of the random guess to ∼20% (114/620). Thus, estimating the site-related properties of the mixed component by their average is viable in our study.

**TABLE 5:** Comparison between the voting and random forest classifiers in model training time, the number of calculated and meta/stable A-mixed oxyhydrides.

Classifiers	F1 score^a^	Training time^b^ (hours)	The number of calculated structures	Meta/stable compounds	Common meta/stable compounds identified by two methods
Random forest	0.84/0.75	6	917	109	102
Voting	0.88/0.80	50	620	114	

a The left or right value refers to the f1 score of classifying the unmixed or mixed compounds, respectively.

b The model training time includes the time of the hyperparameters optimization and feature selection of the two-step ML workflow.

In addition to the oxyhydrides chemistry explored by the informatics, we propose a simple and effective computational workflow targeting the discovery of mixed compounds with huge quantity. Unlike many applications of ML to identify stable materials such as single or double perovskites, a roadblock of our task to identify mixed oxyhydrides mainly lie on two aspects, i.e., the lack of detected database and the rarity of stable oxyhydrides. Even though the HT-DFT methods used to deal with unmixed compounds can generate the small size dataset of the mixed materials by randomly selecting materials, the paucity of stable ones (<5%) can lead to the highly unbalanced data distribution and disable the validity of the database. To handle the issue of insufficient labeled data and unbalanced data distribution, we demonstrate a simple two-step ML and HT-DFT paradigm to identify stable mixed oxyhydrides. The first-round selection based on the ML model transferred from the unmixed ones can provide the valid database of the A-mixed oxyhydrides with a reasonable number of stable labeled compounds. Then, the validated model (f1 score as 0.80) retrained from the mixed materials database can minimize the misclassification of the previous screening. Our workflow demonstrates its efficiency by successfully identifying hundreds of stable mixed oxyhydrides with reasonable cost, which includes all experimentally synthesized materials. In addition to the specific scenario demonstrated in our research, our method consisting of a simple transfer leaning can be applied to many materials design applications, where a large volumes data is unavailable and the data distribution is highly unbalanced.

## 5 Conclusion

We employed comprehensive computations to investigate the stability relationship of the K_2_NiF_4_-type oxyhydrides amid their paucity. The large stability map of the A_2_BH_1+*x*_O_3-*x*_ (*x* = 0, 1, 2) oxyhydrides was constructed by the HT-DFT calculations covering 1856 charge-neutral compositions, with only 5% of the total compounds exhibiting Δ*E*_hull_ ≤ 100 meV/atom. In addition to all laboratory synthesized compounds, our works identified new 76 compositions with good intrinsic thermodynamic stabilities to guide the experimental synthesis. Geometric factors standing for structural distortion and electronegativities accounting for the charge transfer in hydride bonding were unveiled to dominate the oxyhydrides stabilities, and to explain the scarcity of the materials. In addition to the unmixed compounds, we demonstrated an efficient two-step ML workflow consisting of a simple transfer learning to survey the cation-mixed compounds. 114 meta/stable A-mixed oxyhydrides were identified out of total 17618 possible configurations. Facile H^−^ diffusion in the selected compounds indicates the suitability of the oxyhydrides as energy storage and conversion materials. Our study facilitates the discovery of the oxyhydrides, and formulates the computational paradigm for the exploration of other uninvestigated compositional spaces.

## Data Availability

The original contributions presented in the study are included in the article/Supplementary Material, further inquiries can be directed to the corresponding authors.

## References

[B1] Bai Q. He X. Zhu Y. Mo Y. (2018). First-principles study of oxyhydride H– ion conductors: Toward facile anion conduction in oxide-based materials. ACS Appl. Energy Mat. 1 (4), 1626–1634. 10.1021/acsaem.8b00077

[B2] Balachandran P. V. Emery A. A. Gubernatis J. E. Lookman T. Wolverton C. Zunger A. (2018). Predictions of new ABO3 perovskite compounds by combining machine learning and density functional theory. Phys. Rev. Mat. 2 (4), 043802. 10.1103/PhysRevMaterials.2.043802

[B3] Bartel C. J. Sutton C. Goldsmith B. R. Ouyang R. Musgrave C. B. Ghiringhelli L. M. (2019). New tolerance factor to predict the stability of perovskite oxides and halides. Sci. Adv. 5 (2), eaav0693. 10.1126/sciadv.aav0693 30783625 PMC6368436

[B4] Blöchl P. E. (1994). Projector augmented-wave method. Phys. Rev. B 50 (24), 17953–17979. 10.1103/physrevb.50.17953 9976227

[B5] Breiman L. (2001). Random forests. Mach. Learn. 45 (1), 5–32. 10.1023/a:1010933404324

[B6] Bridges C. A. Darling G. R. Hayward M. A. Rosseinsky M. J. (2005). Electronic structure, magnetic ordering, and formation pathway of the transition metal oxide hydride LaSrCoO3H0. 7. J. Am. Chem. Soc. 127 (16), 5996–6011. 10.1021/ja042683e 15839700

[B7] Chen C. Ong S. P. (2021). AtomSets as a hierarchical transfer learning framework for small and large materials datasets. npj Comput. Mat. 7 (1), 173. 10.1038/s41524-021-00639-w

[B8] Chen T. He T. Benesty M. Khotilovich V. Tang Y. Cho H. (2015). Xgboost: Extreme gradient boosting. R. Package Version 0 4-2 1 (4), 1–4.

[B9] Chenebuah E. T. Nganbe M. Tchagang A. B. (2021). Comparative analysis of machine learning approaches on the prediction of the electronic properties of perovskites: A case study of ABX3 and A2BB’X6. Mater. Today Commun. 27, 102462. 10.1016/j.mtcomm.2021.102462

[B10] Cubuk E. D. Sendek A. D. Reed E. J. (2019). Screening billions of candidates for solid lithium-ion conductors: A transfer learning approach for small data. J. Chem. Phys. 150 (21), 214701. 10.1063/1.5093220 31176329

[B11] Emery A. A. Saal J. E. Kirklin S. Hegde V. I. Wolverton C. (2016). High-throughput computational screening of perovskites for thermochemical water splitting applications. Chem. Mat. 28 (16), 5621–5634. 10.1021/acs.chemmater.6b01182

[B12] Ferri F. J. Pudil P. Hatef M. Kittler J. (1994). “Comparative study of techniques for large-scale feature selection,” in Machine intelligence and pattern recognition (Elsevier), 403–413.

[B13] Fjellvåg Ø. S. Nygård K. H. Vajeeston P. Sjåstad A. O. (2019). Advances in the LiCl salt flux method and the preparation of phase pure La2-xNdxLiHO3 (0≤ x≤ 2) oxyhydrides. Chem. Commun. 55 (26), 3817–3820. 10.1039/c9cc00920e 30869684

[B14] Friedman J. H. (2001). Greedy function approximation: A gradient boosting machine. Ann. Statistics 29 (5), 1189–1232. 10.1214/aos/1013203451

[B15] Geurts P. Ernst D. Wehenkel L. (2006). Extremely randomized trees. Mach. Learn. 63 (1), 3–42. 10.1007/s10994-006-6226-1

[B16] Hanaoka K. (2022). Comparison of conceptually different multi-objective Bayesian optimization methods for material design problems. Mater. Today Commun. 31, 103440. 10.1016/j.mtcomm.2022.103440

[B17] Hashimoto W. Tsuji Y. Yoshizawa K. (2020). Optimization of work function via bayesian machine learning combined with first-principles calculation. J. Phys. Chem. C 124 (18), 9958–9970. 10.1021/acs.jpcc.0c01106

[B18] Hayward M. A. Cussen E. J. Claridge J. B. Bieringer M. Rosseinsky M. J. Kiely C. J. (2002). The hydride anion in an extended transition metal oxide array: LaSrCoO3H0. 7. Science 295 (5561), 1882–1884. 10.1126/science.1068321 11884751

[B19] He J. Yao Z. Hegde V. I. Naghavi S. S. Shen J. Bushick K. M. (2020). Computational discovery of stable heteroanionic oxychalcogenides ABXO(A, B= metals; X= S, Se, and Te) and their potential applications. Chem. Mat. 32 (19), 8229–8242. 10.1021/acs.chemmater.0c01902

[B20] He X. Zhu Y. Epstein A. Mo Y. (2018). Statistical variances of diffusional properties from ab initio molecular dynamics simulations. npj Comput. Mat. 4 (1), 18–19. 10.1038/s41524-018-0074-y

[B21] Henkelman G. Arnaldsson A. Jónsson H. (2006). A fast and robust algorithm for bader decomposition of charge density. Comput. Mater. Sci. 36 (3), 354–360. 10.1016/j.commatsci.2005.04.010

[B22] Hoover W. G. (1985). Canonical dynamics: Equilibrium phase-space distributions. Phys. Rev. A . Coll. Park. 31 (3), 1695–1697. 10.1103/physreva.31.1695 9895674

[B23] Iwasaki Y. Matsui N. Suzuki K. Hinuma Y. Yonemura M. Kobayashi G. (2018). Synthesis, crystal structure, and ionic conductivity of hydride ion-conducting Ln2LiHO3 (Ln= La, Pr, Nd) oxyhydrides. J. Mat. Chem. A Mat. 6 (46), 23457–23463. 10.1039/c8ta06880a

[B24] Jain A. Hautier G. Moore C. J. Ong S. P. Fischer C. C. Mueller T. (2011). A high-throughput infrastructure for density functional theory calculations. Comput. Mater. Sci. 50 (8), 2295–2310. 10.1016/j.commatsci.2011.02.023

[B25] Jain A. Ong S. P. Hautier G. Chen W. Richards W. D. Dacek S. (2013). Commentary: The materials Project: A materials genome approach to accelerating materials innovation. Apl. Mater. 1 (1), 011002. 10.1063/1.4812323

[B26] Jha D. Choudhary K. Tavazza F. Liao W. K. Choudhary A. Campbell C. (2019). Enhancing materials property prediction by leveraging computational and experimental data using deep transfer learning. Nat. Commun. 10 (1), 5316. 10.1038/s41467-019-13297-w 31757948 PMC6874674

[B27] Kageyama H. Hayashi K. Maeda K. Attfield J. P. Hiroi Z. Rondinelli J. M. (2018). Expanding frontiers in materials chemistry and physics with multiple anions. Nat. Commun. 9 (1), 772. 10.1038/s41467-018-02838-4 29472526 PMC5823932

[B28] Kobayashi G. Hinuma Y. Matsuoka S. Watanabe A. Iqbal M. Hirayama M. (2016). Pure H- conduction in oxyhydrides. Science 351 (6279), 1314–1317. 10.1126/science.aac9185 26989251

[B29] Kobayashi Y. Hernandez O. Tassel C. Kageyama H. (2017). New chemistry of transition metal oxyhydrides. Sci. Technol. Adv. Mater. 18 (1), 905–918. 10.1080/14686996.2017.1394776 29383042 PMC5784496

[B30] Kobayashi Y. Tsujimoto Y. Kageyama H. (2018). Property engineering in perovskites via modification of anion chemistry. Annu. Rev. Mat. Res. 48, 303–326. 10.1146/annurev-matsci-070317-124415

[B31] König G. Molnar C. Bischl B. Grosse-Wentrup M. (2021). “Relative feature importance,” in 2020 25th International Conference on Pattern Recognition (ICPR), Milan, Italy, 10-15 January 2021 (IEEE), 9318–9325. 10.1109/ICPR48806.2021.9413090

[B32] Kresse G. Furthmüller J. (1996). Efficient iterative schemes for *ab initio* total-energy calculations using a plane-wave basis set. Phys. Rev. B 54 (16), 11169–11186. 10.1103/physrevb.54.11169 9984901

[B33] Kumari S. Kumar D. Mittal M. (2021). An ensemble approach for classification and prediction of diabetes mellitus using soft voting classifier. Int. J. Cognitive Comput. Eng. 2, 40–46. 10.1016/j.ijcce.2021.01.001

[B34] Lavén R. Haussermann U. Perrichon A. Andersson M. S. Targama M. S. Demmel F. (2021). Diffusional dynamics of hydride ions in the layered oxyhydride SrVO2H. Chem. Mat. 33 (8), 2967–2975. 10.1021/acs.chemmater.1c00505 PMC8154327 34054217

[B35] Li W. Ionescu E. Riedel R. Gurlo A. (2013). Can we predict the formability of perovskite oxynitrides from tolerance and octahedral factors? J. Mat. Chem. A Mat. 1 (39), 12239. 10.1039/c3ta10216e

[B36] Liu H. Feng J. Dong L. (2022). Quick screening stable double perovskite oxides for photovoltaic applications by machine learning. Ceram. Int. 48 (13), 18074–18082. 10.1016/j.ceramint.2022.02.258

[B37] Liu X. Bjørheim T. S. Vines L. Fjellvåg Ø. S. Granerød C. Prytz Ø. (2019). Highly correlated hydride ion tracer diffusion in SrTiO_3–x_H_x_ oxyhydrides. J. Am. Chem. Soc. 141 (11), 4653–4659. 10.1021/jacs.8b12985 30802045

[B38] Maeda K. Takeiri F. Kobayashi G. Matsuishi S. Ogino H. Ida S. (2022). Recent progress on mixed-anion materials for energy applications. Bull. Chem. Soc. Jpn. 95 (1), 26–37. 10.1246/bcsj.20210351

[B39] Maintz S. Deringer V. L. Tchougréeff A. L. Dronskowski R. (2016). Lobster: A tool to extract chemical bonding from plane‐wave based DFT. J. Comput. Chem. 37 (11), 1030–1035. 10.1002/jcc.24300 26914535 PMC5067632

[B40] Masuda N. Kobayashi Y. Hernandez O. Bataille T. Paofai S. Suzuki H. (2015). Hydride in BaTiO2. 5H0. 5: A labile ligand in solid state chemistry. J. Am. Chem. Soc. 137 (48), 15315–15321. 10.1021/jacs.5b10255 26575595

[B41] Matsui N. Hinuma Y. Iwasaki Y. Suzuki K. Guangzhong J. Nawaz H. (2020). The effect of cation size on hydride-ion conduction in LnSrLiH2O2 (Ln= La, Pr, Nd, Sm, Gd) oxyhydrides. J. Mat. Chem. A Mat. 8 (46), 24685–24694. 10.1039/d0ta06728h

[B42] Mikita R. Aharen T. Yamamoto T. Takeiri F. Ya T. Yoshimune W. (2016). Topochemical nitridation with anion vacancy-assisted N3–/O2– exchange. J. Am. Chem. Soc. 138 (9), 3211–3217. 10.1021/jacs.6b00088 26855196

[B43] Mo Y. Ong S. P. Ceder G. (2011). First principles study of the Li10GeP2S12 lithium super ionic conductor material. Chem. Mat. 24 (1), 15–17. 10.1021/cm203303y

[B44] Nawaz H. Takeiri F. Kuwabara A. Yonemura M. Kobayashi G. (2020). Synthesis and H− conductivity of a new oxyhydride Ba2YHO3 with anion-ordered rock-salt layers. Chem. Commun. 56 (71), 10373–10376. 10.1039/d0cc03638b 32766634

[B45] Nelson R. Ertural C. George J. Deringer V. L. Hautier G. Dronskowski R. (2020). LOBSTER: Local orbital projections, atomic charges, and chemical‐bonding analysis from projector‐augmented‐wave‐based density‐functional theory. J. Comput. Chem. 41 (21), 1931–1940. 10.1002/jcc.26353 32531113

[B46] Nosé S. (1984). A unified formulation of the constant temperature molecular dynamics methods. J. Chem. Phys. 81 (1), 511–519. 10.1063/1.447334

[B47] Ong S. P. Wang L. Kang B. Ceder G. (2008). Li−Fe−P−O2 phase diagram from first principles calculations. Chem. Mat. 20 (5), 1798–1807. 10.1021/cm702327g

[B48] Ouyang B. Wang J. He T. Bartel C. J. Huo H. Wang Y. (2021). Synthetic accessibility and stability rules of NASICONs. Nat. Commun. 12, 5752. 10.1038/s41467-021-26006-3 34599170 PMC8486869

[B49] Pedregosa F. Varoquaux G. Gramfort A. Michel V. Thirion B. Grisel O. (2011). Scikit-learn: Machine learning in Python. J. Mach. Learn. Res. 12, 2825–2830.

[B50] Perdew J. P. Burke K. Ernzerhof M. (1996). Generalized gradient approximation made simple. Phys. Rev. Lett. 77 (18), 3865–3868. 10.1103/physrevlett.77.3865 10062328

[B51] Pudil P. Novovičová J. Kittler J. (1994). Floating search methods in feature selection. Pattern Recognit. Lett. 15 (11), 1119–1125. 10.1016/0167-8655(94)90127-9

[B52] Shen J. Hegde V. I. He J. Xia Y. Wolverton C. (2021). High-throughput computational discovery of ternary mixed-anion oxypnictides. Chem. Mat. 33 (24), 9486–9500. 10.1021/acs.chemmater.1c02294

[B53] Shi L. Chang D. Ji X. Lu W. (2018). Using data mining to search for perovskite materials with higher specific surface area. J. Chem. Inf. Model. 58 (12), 2420–2427. 10.1021/acs.jcim.8b00436 30457872

[B54] Sun W. Bartel C. J. Arca E. Bauers S. R. Matthews B. Orvañanos B. (2019). A map of the inorganic ternary metal nitrides. Nat. Mat. 18 (7), 732–739. 10.1038/s41563-019-0396-2 31209391

[B55] Sun W. Dacek S. T. Ong S. P. Hautier G. Jain A. Richards W. D. (2016). The thermodynamic scale of inorganic crystalline metastability. Sci. Adv. 2 (11), e1600225. 10.1126/sciadv.1600225 28138514 PMC5262468

[B56] Takeiri F. Watanabe A. Kuwabara A. Nawaz H. Ayu N. I. P. Yonemura M. (2019). Ba2ScHO3: H– conductive layered oxyhydride with H– site selectivity. Inorg. Chem. 58 (7), 4431–4436. 10.1021/acs.inorgchem.8b03593 30784265

[B57] Takeiri F. Watanabe A. Okamoto K. Bresser D. Lyonnard S. Frick B. (2022). Hydride-ion-conducting K2NiF4-type Ba-Li oxyhydride solid electrolyte. Nat. Mat. 21, 325–330. 10.1038/s41563-021-01175-0 35027719

[B58] Talapatra A. Uberuaga B. P. Stanek C. R. Pilania G. (2021). A machine learning approach for the prediction of formability and thermodynamic stability of single and double perovskite oxides. Chem. Mat. 33 (3), 845–858. 10.1021/acs.chemmater.0c03402

[B59] Tang W. Sanville E. Henkelman G. (2009). A grid-based bader analysis algorithm without lattice bias. J. Phys. Condens. Matter 21 (8), 084204. 10.1088/0953-8984/21/8/084204 21817356

[B60] Tao Q. Xu P. Li M. Lu W. (2021). Machine learning for perovskite materials design and discovery. npj Comput. Mat. 7 (1), 23–18. 10.1038/s41524-021-00495-8

[B61] Tassel C. Goto Y. Watabe D. Tang Y. Lu H. Kuno Y. (2016). High‐pressure synthesis of manganese oxyhydride with partial anion order. Angew. Chem. Int. Ed. Engl. 55 (33), 9819–9822. 10.1002/ange.201605123 27355695

[B62] Wang H.-C. Schmidt J. Botti S. Marques M. A. L. (2021). A high-throughput study of oxynitride, oxyfluoride and nitrofluoride perovskites. J. Mat. Chem. A Mat. 9 (13), 8501–8513. 10.1039/d0ta10781f

[B63] Yajima T. Takahashi K. Nakajima H. Honda T. Ikeda K. Otomo T. (2022). High-pressure synthesis of transition-metal oxyhydrides with double-perovskite structures. Inorg. Chem. 61 (4), 2010–2016. 10.1021/acs.inorgchem.1c03162 35034444

[B64] Yajima T. Takeiri F. Aidzu K. Akamatsu H. Fujita K. Yoshimune W. (2015). A labile hydride strategy for the synthesis of heavily nitridized BaTiO3. Nat. Chem. 7 (12), 1017–1023. 10.1038/nchem.2370 26587718

[B65] Yamaguchi S. (2016). Large, soft, and polarizable hydride ions sneak around in an oxyhydride. Science 351 (6279), 1262–1263. 10.1126/science.aaf3361 26989234

[B66] Ye W. Chen C. Wang Z. Chu I. H. Ong S. P. (2018). Deep neural networks for accurate predictions of crystal stability. Nat. Commun. 9 (1), 3800. 10.1038/s41467-018-06322-x 30228262 PMC6143552

[B67] Zapp N. Sheptyakov D. Kohlmann H. (2021). Computational chemistry-guided syntheses and crystal structures of the heavier lanthanide hydride oxides DyHO, ErHO, and LuHO. Crystals 11 (7), 750. 10.3390/cryst11070750

[B68] Zhang H. Li N. Li K. Xue D. (2007). Structural stability and formability of ABO3-type perovskite compounds. Acta Crystallogr. B 63 (6), 812–818. 10.1107/s0108768107046174 18004035

