# Supplementary material for: Computation-accelerated discovery of the K2NiF4-type oxyhydrides combing density functional theory and machine learning approach
Source: Front Chem. 2022 Aug 26;10:964953. doi: 10.3389/fchem.2022.964953 (PMC9458981; doi:10.3389/fchem.2022.964953)
Supplement: Supplementary file 1 [file Presentation1.pdf]

Supplementary Material

Computation-Accelerated Discovery of the K_2NiF_4-type Oxyhydrides combing Density Function Theory and Machine Learning Approach

Qiang Bai, * Yunrui Duan, Jie Lian and Xiaomin Wang*

College of Materials Science and Engineering, Taiyuan University of Technology, Taiyuan, 030024, China

*Email: baiqiang@tyut.edu.cn, wangxiaomin@tyut.edu.cn

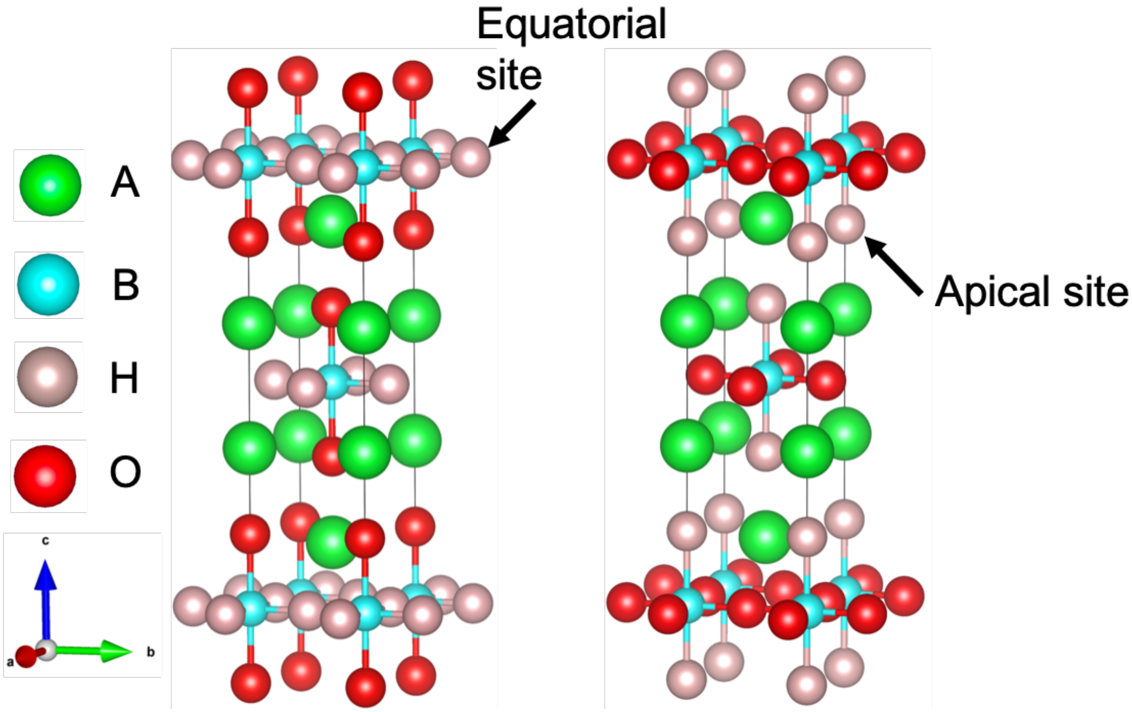

Figure S1. The preference of H^- over (a) equatorial sites and (b) apical sites in the $A_2BH_{1+x}O_{3-x}$ oxyhydrides, respectively.

Table S1. 25 features for the permutation-based importance evaluation.

Elemental features					
Symbol	Description	Symbol	Description	Symbol	Description
χ_A	Pauling's electronegativity of element at A site	r_B	Shannon ionic radius for ion at B site	χ_B	Pauling's electronegativity of element at B site
M_A	Mendeleev number of an element at A site	I_{1A}	First ionization energy of element at A site	r_A	Shannon ionic radius for ion at A site
I_{1B}	First ionization energy of element at B site	e^- affinity$_B$	Electron affinity of element at B site	Z_A	Atomic number of an element at A site
group B	Group number of an element at B site	Z_B	Atomic number of an element at B site	row B	Row number of an element at B site
atomic weight$_A$	Atomic weight of atom at A site	group A	Group number of an element at A site	atomic weight$_B$	Atomic weight of atom at A site
M_B	Mendeleev number of an element at B site	row A	Row number of an element at A site	valence e^-_B	Number of valence electrons of atom at B site
e^- affinity$_A$	Electron affinity of element at A site	valence e^-_A	Electrons of atom at A site		
**Compositional and structural features**					
Symbol	Description	Symbol	Description	Symbol	Description
t_{bv}	$\frac{d_{A-X}}{\sqrt{2}d_{B-X}}$	H content	Number of H in composition	Oxidation state$_B$	Oxidation state of element at B site
Oxidation state$_A$	Oxidation state of element at B site	O content	Number of O in composition		

Table S2. Elemental, compositional and structural features of the oxyhydrides for machine learning.

Elemental features					
Symbol	Description	Symbol	Description	Symbol	Description
χ_A	Pauling's electronegativity of element at A site	χ_B	Pauling's electronegativity of element at B site	r_A	Shannon ionic radius for ion at A site
r_B	Shannon ionic radius for ion at B site	EA_A	Electron affinity of element at A site	EA_B	Electron affinity of element at B site
χ_A-χ_B	χ_A-χ_B	χ_{avg}	$(\chi_A+\chi_B)/2$	χ_{std}	Standard deviation between χ_A and χ_B
r_{avg}	$(r_A+r_B)/2$	r_{std}	Standard deviation between r_A and r_B	r_A-r_B	r_A-r_B
EA_A-EA_B	EA_A-EA_B	EA_{avg}	$(EA_A+EA_B)/2$	EA_{std}	Standard deviation between EA_A and EA_B
Row A	Row number of an element at A site	Row B	Row number of an element at B site	Group A	Group number of an element at A site
Group B	Group number of an element at B site	I_{1A}	First ionization energy of element at A site	I_{1B}	First ionization energy of element at B site
M_A	Mendeleev number of an element at A site	M_B	Mendeleev number of an element at B site	Z_A	Atomic number of an element at A site
Z_B	Atomic number of an element at B site	AIR_A	Average ionic radius of ion at A site	AIR_B	Average ionic radius of ion at B site
ACR_A	Average cationic radius of ion at A site	ACR_B	Average cationic radius of ion at B site	χ'_A	Allred-Rochow electronegativity of element at A site
χ'_B	Allred-Rochow electronegativity of element at B site	d_{0OA}	d_0 from bond valence parameter between O^{2-} and the specie at A site	d_{0OB}	d_0 from bond valence parameter between O^{2-} and the specie at B site
CR_A	Covalent radius of element at A site	CR_B	Covalent radius of element at B site	AR_A	Atomic radius of element at A site
AR_B	Atomic radius of element at B site	VR_A	Van der Waals radius of element at A site	VR_B	Van der Waals radius of element at B site
AV_A	Atomic volume of atom at A site	AV_B	Atomic volume of atom at B site	DP_A	Dipole polarizability of element at A site
DP_B	Dipole polarizability of element at B site	AW_A	Atomic weight of atom at A site	AW_B	Atomic weight of atom at B site
NV_A	Number of valence electrons of atom at A site	NV_B	Number of valence electrons of atom at B site	d_{0HA}	d_0 from bond valence parameter between H^- and the specie at A site
d_{0HB}	d_0 from bond valence parameter between H^- and the specie at B site				

Compositional and structural features					
Symbol	Description	Symbol	Description	Symbol	Description
t_o	$\frac{r_A + r_O}{\sqrt{2}(r_B + r_O)}$	Oxi_A	Oxidation state of element at A site	Oxi_B	Oxidation state of element at B site
Oxi_A-Oxi_B	Oxi_A-Oxi_B	Oxi_{avg}	$(Oxi_A+Oxi_B)/2$	Oxi_{std}	Standard deviation of Oxi_A and Oxi_B

t_{bv}	$\frac{d_{A-X}}{\sqrt{2}d_{B-X}}$	t_{bvO}	$\frac{d_{A-O}}{\sqrt{2}d_{B-O}}$	d_{A-O}	$d_{0OA} - b_{0OA} \times ln\frac{Oxi_A}{CN(A)}$
d_{B-O}	$d_{0OB} - b_{0OAB} \times ln\frac{Oxi_B}{CN(B)}$	τ	$\frac{r_X}{r_B} - n_A(n_A - \frac{\frac{r_A}{r_B}}{\ln\left(\frac{r_A}{r_B}\right)})$	t_{bvH}	$\frac{d_{A-H}}{\sqrt{2}d_{B-H}}$
d_{A-H}	$d_{0HA} - b_{0HA} \times ln\frac{Oxi_A}{CN(A)}$	d_{B-H}	$d_{0HB} - b_{0HB} \times ln\frac{Oxi_B}{CN(B)}$	N_H	Number of H in composition
N_O	Number of O in composition	d_{A-X}	d_{A-X} that satisfies $Oxi_A = CN(A-O) \times e^{\frac{d_{0OA}-d_{A-X}}{b_{0OA}}} + CN(A-H) \times e^{\frac{d_{0HA}-d_{A-X}}{b_{0HA}}}$	d_{B-X}	d_{B-X} that satisfies $Oxi_B = CN(B-O) \times e^{\frac{d_{0HB}-d_{B-X}}{b_{0HB}}} + CN(B-H) \times e^{\frac{d_{0HB}-d_{B-X}}{b_{0HB}}}$
$(d_{A-X})_{avg}$	(CN(A-O) ×d_{A-O} +CN(A-H)×d_{A-H})/(CN(A-O)+CN(A-H))	$(d_{B-X})_{avg}$	CN(B-O) ×d_{B-O} +CN(B-H) ×d_{B-H})/(CN(B-O) +CN(B-H))	t_{avg}	$\frac{(d_{A-X})_{avg}}{\sqrt{2}(d_{B-X})_{avg}}$

Note S1. The voting classifier[1] trains on an ensemble of numerous conceptually different classifiers to predict an output based on their average predicted probabilities. Results with the highest average predicted probabilities upon voting will be returned by the voting model. The ensemble of models contributing to the voting classifiers in our paper are extra trees, random forest, SVC, XGBoost and Gradient boosting classifiers. The voting classifier can balance out the weakness of an individual model and may yield a good accuracy.

Note S2. Multiple metrics can evaluate performance of classifiers, which are defined as:

$$\mathrm{accuracy} = \frac{\mathrm{TP} + \mathrm{TN}}{\mathrm{TP} + \mathrm{TN} + \mathrm{FP} + \mathrm{FN}} \quad (\mathrm{S1}),$$

$$\mathrm{precision} = \frac{\mathrm{TP}}{\mathrm{TP} + \mathrm{FP}} \quad (\mathrm{S2}),$$

$$\mathrm{recall} = \frac{\mathrm{TP}}{\mathrm{TP} + \mathrm{FN}} \quad (\mathrm{S3}),$$

$$\mathrm{f1\ score} = \frac{2 \times \mathrm{precision} \times \mathrm{recall}}{\mathrm{precision} + \mathrm{recall}} \quad (\mathrm{S4}),$$

$$\mathrm{specificity} = \frac{\mathrm{TN}}{\mathrm{TN} + \mathrm{FP}} \quad (\mathrm{S5}),$$

where TP, TN, FP, FN indicate true positive, true negative, false positive and false negative, respectively. The receiver operating characteristic (ROC) curve describes a relationship between the recall and 1- specificity as a function of different probability thresholds given by the model. The precision-recall curve describes a relationship between precision and recall as a function of different probability thresholds given by the model.

Note S3. The sequential forward floating selection (SFFS)[2, 3] is a greedy search algorithm that aims to find a subset of features most relevant to the problem out of the whole feature space. The SFFS starts with an empty feature set and adds one feature per step to achieve the best model performance (i.e., the f1 score in our case) at the corresponding feature dimensionality. The floating variant in the SFFS corresponds to a following conditional exclusion step to drop one feature already included, if the model performance can be improved by the removal. The inclusion and exclusion of features repeat until the desired feature quantity is achieved. The difference between SFFS and recursive feature elimination (RFE) is the criterion to select features. RFE eliminates features in terms of the feature importance or the weight coefficients, whereas SFFS selects features based on the model performance metric (e.g., accuracy, f1 score, precision) defined by users. Thus, SFFS is more complex than RFE.

Table S3. Selected features to classify the oxyhydrides using different classifiers.

Classifiers	Selected features
Voting	χ_{avg}, Oxi_{avg}, t_o, M_A, Z_B, AIR_B, $d_{B\text{-}O}$, AV_B, AW_B, NV_B, $d_{B\text{-}X}$, t_{bv}, $d_{B\text{-}H}$
Extra trees	χ_B, r_B, EA_A, EA_B, Oxi_A, Oxi_B, χ_A-χ_B, χ_{avg}, χ_{std}, r_A-r_B, r_{avg}, r_{std}, EA_A-EA_B, EA_{std}, Oxi_A-Oxi_B, Oxi_{avg}, Oxi_{std}, I_{1B}, t_o, M_A, M_B, Z_B, Row B, AIR_B, ACR_B, $d_{A\text{-}O}$, $d_{B\text{-}O}$, CR_A, CR_B, χ'_B, AR_B, VR_B, AV_B, AV_A, DP_A, t_{bvO}, τ , NV_B, N_O, $d_{B\text{-}X}$, t_{bv}, d_{0HB}, $d_{B\text{-}X}$, t_{bvH}, $(d_{B\text{-}X})_{avg}$
Random forest	χ_B, r_A, EA_A, EA_B, Oxi_A, χ_{avg}, χ_{std}, EA_A-EA_B, EA_{avg}, EA_{std}, Oxi_A-Oxi_B, Oxi_{std}, I_{1B}, t_o, M_B, Group A, $d_{B\text{-}O}$, CR_B, AR_A, AR_B, VR_A, VR_B, AV_A, AV_B, DP_A, DP_B, AW_B, NV_A, NV_B, N_O, $d_{A\text{-}X}$, $d_{B\text{-}X}$, t_{bv}, d_{0HB}, $d_{B\text{-}H}$, $(d_{B\text{-}X})_{avg}$
SVC	χ_{avg}, r_A-r_B, Oxi_{std}, t_o, M_A, Group B, AIR_B, $d_{A\text{-}O}$, CR_B, AV_B, NV_A, t_{bv}
XGBoost	χ_B, r_B, EA_B, Oxi_A, Oxi_B, χ_A-χ_B, χ_{avg}, EA_A-EA_B, EA_{avg}, EA_{std}, Oxi_A-Oxi_B, Oxi_{avg}, Oxi_{std}, I_{1B}, t_o, Z_A, Z_B, Row A, Row B, Group B, $d_{A\text{-}O}$, d_{0OB}, $d_{B\text{-}O}$, CR_B, AR_B, VR_B, DP_B, AW_A, AW_B, t_{bvO}, NV_B, N_H, N_O, $d_{A\text{-}X}$, $d_{B\text{-}X}$, t_{bv}, d_{0HA}, d_{0HB}, $d_{B\text{-}H}$, $(d_{A\text{-}X})_{avg}$, $(d_{B\text{-}X})_{avg}$
Gradient boosting	χ_B, EA_B, χ_A-χ_B, χ_{avg}, χ_{std}, r_{std}, EA_A-EA_B, EA_{avg}, EA_{std}, Oxi_{avg}, I_{1B}, t_o, M_B, Z_B, Row B, Group B, AIR_B, ACR_B, $d_{B\text{-}O}$, CR_B, AR_B, VR_B, AV_B, DP_B, AW_B, t_{bvO}, $d_{B\text{-}X}$, t_{bv}, d_{0HB}, $d_{B\text{-}H}$, $(d_{B\text{-}X})_{avg}$
Decision tree	χ_A, χ_B, r_A, r_B, EA_A, EA_B, χ_{avg}, χ_{std}, r_{avg}, r_{std}, r_A-r_B, EA_A-EA_B, EA_{avg}, EA_{std}, Row A, Row B, Group A, Group B, I_{1A}, I_{1B}, M_A, M_B, Z_A, Z_B, AIR_A, AIR_B, ACR_A, ACR_B, χ'_A, χ'_B, d_{0OA}, d_{0OB}, CR_A, CR_B, AR_A, AR_B, VR_A, VR_B, AV_A, AV_B, DP_A, DP_B, AW_A, AW_B, NV_A, NV_B, d_{0HA}, d_{0HB}, t_o, Oxi_A, Oxi_B, Oxi_A-Oxi_B, Oxi_{avg}, Oxi_{std}, t_{bv}, t_{bvO}, $d_{A\text{-}O}$, $d_{B\text{-}O}$, τ, t_{bvH}, $d_{A\text{-}H}$, $d_{B\text{-}H}$, N_H, N_O, $d_{A\text{-}X}$, $d_{B\text{-}X}$, $(d_{A\text{-}X})_{avg}$, $(d_{B\text{-}X})_{avg}$, t_{avg}

Table S4. Computation time of the model training including hyperparameters optimization and feature selection trained by the unmixed oxyhydrides (first round machine learning).

Classifiers	Time of hyperparameters optimization (s)	Time of feature selection (s)	Total time (s)
Extra trees	2600	8040	10640
Random forest	2344	10303	12647
SVC	1639	3379	5018
XGBoost	6829	24738	31567
Gradient boosting	4244	21882	26126
Decision tree	33	461	494
Voting	17549	92113	109662

Table S5. Computation time of the model training including hyperparameters optimization and feature selection trained by the mixed oxyhydrides (second round machine learning).

Classifiers	Time of hyperparameters optimization (s)	Time of feature selection (s)	Total time (s)
Random forest	1804	5731	7535
Voting	15202	55380	70582

Table S6. Optimized hyperparameters and the search space of different classifiers trained based on the unmixed oxyhydrides, i.e., the first ML step.

Classifiers	Optimized parameters	Search space in GridSearchCV
Extra trees	critertion: gini; max_depth: 12; max_featurers: sqrt; min_samples_leaf: 1; n_estimators: 500	criterion: [gini, entropy]; max_depth: range (1,15); max_features: [None, log2, sqrt]; min_samples_leaf: [1, 0.01, 0.001]; n_estimators: range (25, 525, 25)
Random forest	critertion: entropy; max_depth: 11; max_featurers: sqrt; min_samples_leaf: 0.001; n_estimators: 50	criterion: [gini, entropy]; max_depth: range (1,15); max_features: [None, log2, sqrt]; min_samples_leaf: [1, 0.01, 0.001]; n_estimators: range (25, 525, 25)
SVC	kernel: rbf; gamma: 0.3; C: 10	kernel: [linear, rbf, poly]; C: [0.1,1,10, 100, 1000]; gamma: range (0.1, 1, 0.1)
XGBoost	max_depth: 8; n_estimators: 50; colsample_bytree: 0.8; gamma: 2; min_child_weight: 1; subsample: 0.6	max_depth: [2, 4, 6, 8, 10]; n_estimators: [50, 100, 200, 300, 400, 500]; colsample_bytree: [0.6, 0.8, 1.0]; gamma: [0.5, 1, 1.5, 2, 5]; min_child_weight: [1, 5, 10]; subsample: [0.6, 0.8, 1.0]
Gradient boosting	loss: deviance; critertion: mae; max_depth: 9; max_featurers: sqrt; min_samples_leaf: 4; n_estimators: 300; subsample: 0.6; learning_rate: 0.1	loss: [deviance; exponential]; criterion: [mae, friedman_mse]; max_depth: [3, 5, 7, 9]; max_features: [None, log2, sqrt]; min_samples_leaf: [4, 5, 6]; n_estimators: [5, 50, 100, 200, 300, 400, 500]; subsample: [0.6, 0.7, 0.8]; learning_rate: [0.01, 0.05, 0.1,0.5, 1]
Decision tree	critertion: gini; max_depth: 4; max_featurers: None; min_samples_leaf: 1; min_samples_split: 2	criterion: [gini, entropy]; max_depth: range (1,20); max_features: [None, log2, sqrt]; min_samples_leaf: range (1, 5); min_samples_split: range (2,10)
Voting	Voting: soft<br><br>Extra trees: critertion: entropy; max_depth: 13; max_featurers: sqrt; min_samples_leaf: 1; n_estimators: 75<br><br>Random forest: critertion: entropy; max_depth: 14; max_featurers: sqrt; min_samples_leaf: 1; n_estimators: 25<br><br>SVC: kernel: rbf; C: 10; gamma=0.2<br><br>XGBoost: max_depth: 4; n_estimators: 300; colsample_bytree: 1.0; gamma: 1.5; min_child_weight: 1; subsample: 0.6<br><br>Gradient boosting: loss: deviance; critertion: friedman_mse; max_depth: 9; max_featurers: log2; min_samples_leaf: 4; n_estimators: 400; subsample: 0.7; learning_rate: 0.05	Same as each constituting classifier

Table S7. Optimized hyperparameters of different classifiers trained by the A-mixed oxyhydrides, i.e., the second ML step.

Classifiers	Optimized parameters
Random forest	critertion: entropy; max_depth: 8; max_featurers: None; min_samples_leaf: 1; n_estimators: 125
Voting	Voting: soft<br><br>Extra trees: critertion: entropy; max_depth: 10; max_featurers: None; min_samples_leaf: 1; n_estimators: 50<br><br>Random forest: critertion: gini; max_depth: 6; max_featurers: None; min_samples_leaf: 1; n_estimators: 225<br><br>SVC: kernel: poly; gamma: 0.2; C: 1000; degree: 3<br><br>XGBoost: max_depth: 6; n_estimators: 200; colsample_bytree: 1.0; gamma: 2; min_child_weight: 1; subsample: 0.6<br><br>Gradient boosting: loss: exponential; critertion: friedman_mse; max_depth: 9; max_featurers: sqrt; min_samples_leaf: 5; n_estimators: 200; subsample: 0.6; learning_rate: 0.05

Table S8. The predicted unmixed oxyhydrides with $\Delta E_{hull} \leq 100$ meV/atom and their Bader charge of H. The previously known compounds are highlighted in bold.

Composition	ΔE_{hull} (meV/atom)	Bader charge of H (e^-)	Composition	ΔE_{hull} (meV/atom)	Bader charge of H (e^-)
Na_2NbHO_3	74	-0.69	Na_2TaHO_3	40	-0.71
K_2NbHO_3	0	-0.65	K_2TaHO_3	19	-0.66
Rb_2NbHO_3	29	-0.64	Rb_2TaHO_3	37	-0.65
Cs_2NbHO_3	95	-0.63	Cs_2TaHO_3	98	-0.64
Tl_2PHO_3	80	-1.00	Ca_2AlHO_3	39	-0.75
Ca_2ScHO_3	98	-0.71	Ca_2CrHO_3	51	-0.53
Ca_2GaHO_3	84	-0.55	Sr_2AlHO_3	33	-0.73
Sr_2ScHO_3	28	-0.72	Sr_2CrHO_3	0	-0.53
Sr_2GaHO_3	22	-0.54	**Ba_2ScHO_3**	0	-0.70
Ba_2CrHO_3	54	-0.66	Ba_2GaHO_3	54	-0.52
**Ba_2YHO_3**	65	-0.71	Ba_2InHO_3	63	-0.51
Ba_2SmHO_3	100	-0.51	Ba_2TbHO_3	65	-0.71
Ba_2DyHO_3	65	-0.71	Ba_2HoHO_3	49	-0.71
Ba_2TmHO_3	34	-0.71	Ba_2LuHO_3	25	-0.71
Eu_2AlHO_3	32	-0.74	Eu_2ScHO_3	67	-0.70
Eu_2CrHO_3	0	-0.58	Eu_2FeHO_3	63	-0.53
Eu_2GaHO_3	12	-0.53	Y_2LiHO_3	100	-0.67
**La_2LiHO_3**	20	-0.64	Ce_2LiHO_3	34	-0.62
**Pr_2LiHO_3**	11	-0.66	**Nd_2LiHO_3**	21	-0.66
**Sm_2LiHO_3**	43	-0.66	Gd_2LiHO_3	66	-0.64
Tb_2LiHO_3	83	-0.67	Dy_2LiHO_3	96	-0.67
Ba_2ErHO_3	50	-0.71	$Na_2TiH_2O_2$	58	-0.66
$Na_2ZrH_2O_2$	82	-0.69	$Na_2HfH_2O_2$	65	-0.72
$K_2TiH_2O_2$	56	-0.63	$K_2ZrH_2O_2$	37	-0.67
$K_2HfH_2O_2$	33	-0.66	$Rb_2ZrH_2O_2$	57	-0.67
$Rb_2HfH_2O_2$	57	-0.65	$Cs_2ZrH_2O_2$	94	-0.65
$Cs_2HfH_2O_2$	97	-0.66	$Ca_2MnH_2O_2$	94	-0.65
$Sr_2MgH_2O_2$	62	-0.78	$Sr_2MnH_2O_2$	13	-0.64
$Sr_2ZnH_2O_2$	14	-0.55	$Ba_2MgH_2O_2$	33	-0.74
$Ba_2MnH_2O_2$	0	-0.60	$Ba_2ZnH_2O_2$	26	-0.53
$Eu_2MgH_2O_2$	87	-0.76	$Eu_2CoH_2O_2$	2	-0.52
$Eu_2ZnH_2O_2$	13	-0.53	$Ca_2ZnH_2O_2$	70	-0.54
Li_2AlH_3O	99	-0.95	Na_2AlH_3O	61	-0.93
Na_2ScH_3O	89	-0.72	Na_2CrH_3O	73	-0.62
K_2AlH_3O	78	-0.88	K_2ScH_3O	43	-0.69
K_2CrH_3O	63	-0.59	K_2LuH_3O	96	-0.72
Rb_2AlH_3O	99	-0.88	Rb_2ScH_3O	56	-0.68
Rb_2CrH_3O	87	-0.58	Rb_2LuH_3O	97	-0.71
Cs_2AlH_3O	50	-0.87	Cs_2ScH_3O	81	-0.67
Hg_2PH_3O	95	-1.04	Ca_2LiH_3O	26	-0.74
**Sr_2LiH_3O**	0	-0.74	**Ba_2LiH_3O**	0	-0.72
Ba_2NaH_3O	22	-0.71	Eu_2LiH_3O	48	-0.71

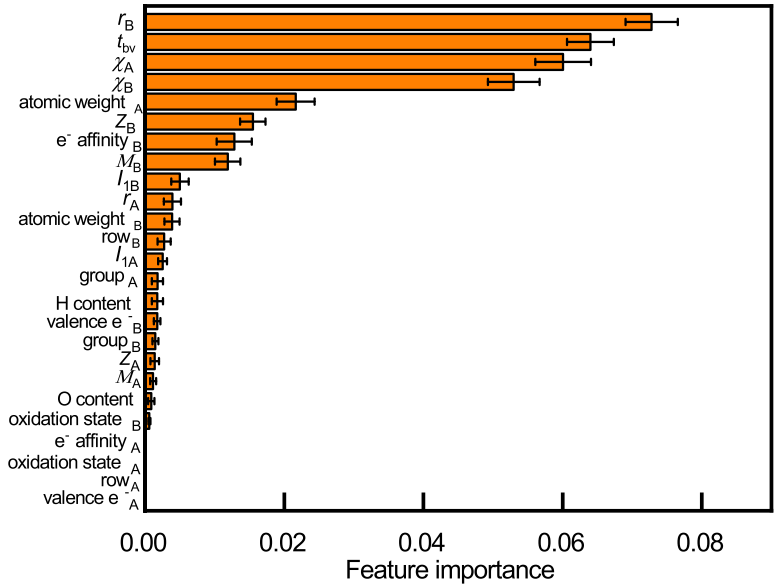

Figure S2. Feature importance to classify oxyhydrides stability based on the feature importance using the decision tree model.

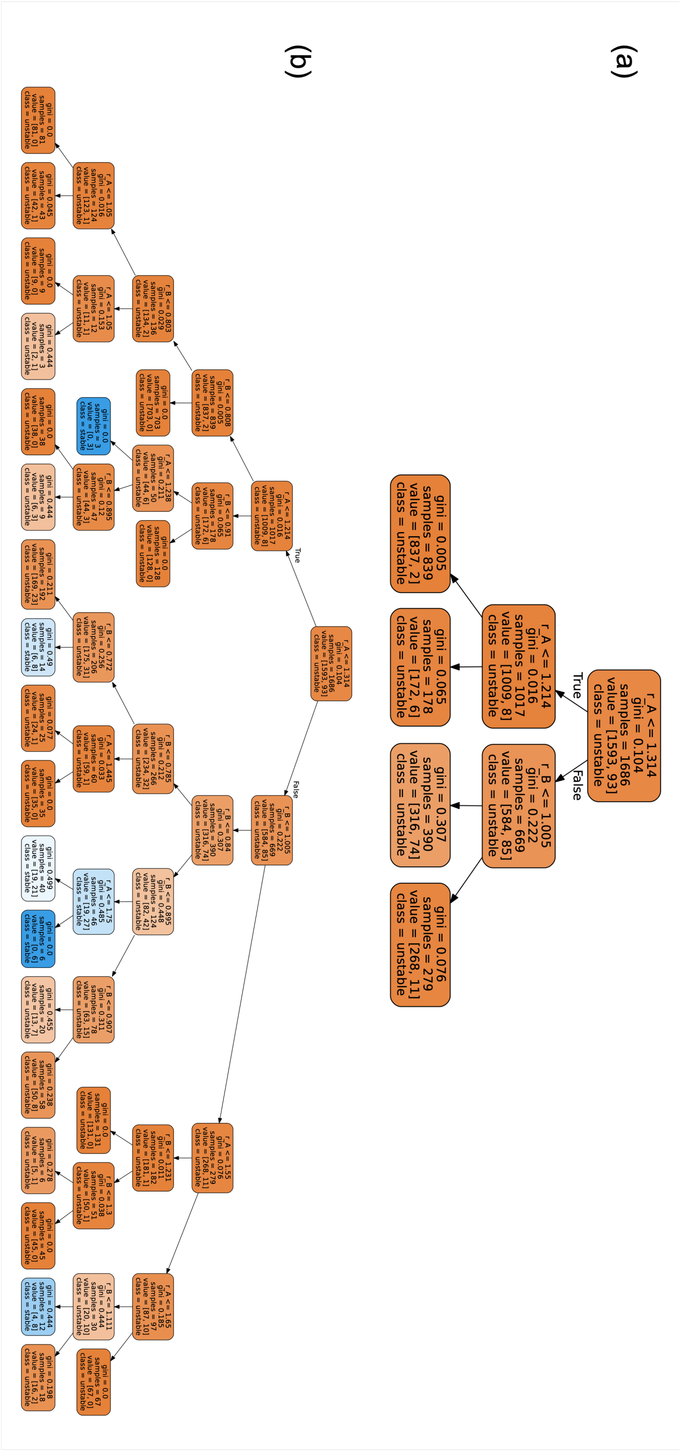

Figure S3. Analysis of r_a and r_b to differential meta/stable oxyhydrides using the decision tree with the max depth as (a) 2 and (b) 5, respectively. The blue boxes correspond to the stable categories, and the orange boxes refer to the unstable categories. The value in each box indicates the number of unstable (left) and stable (right) compounds, respectively.

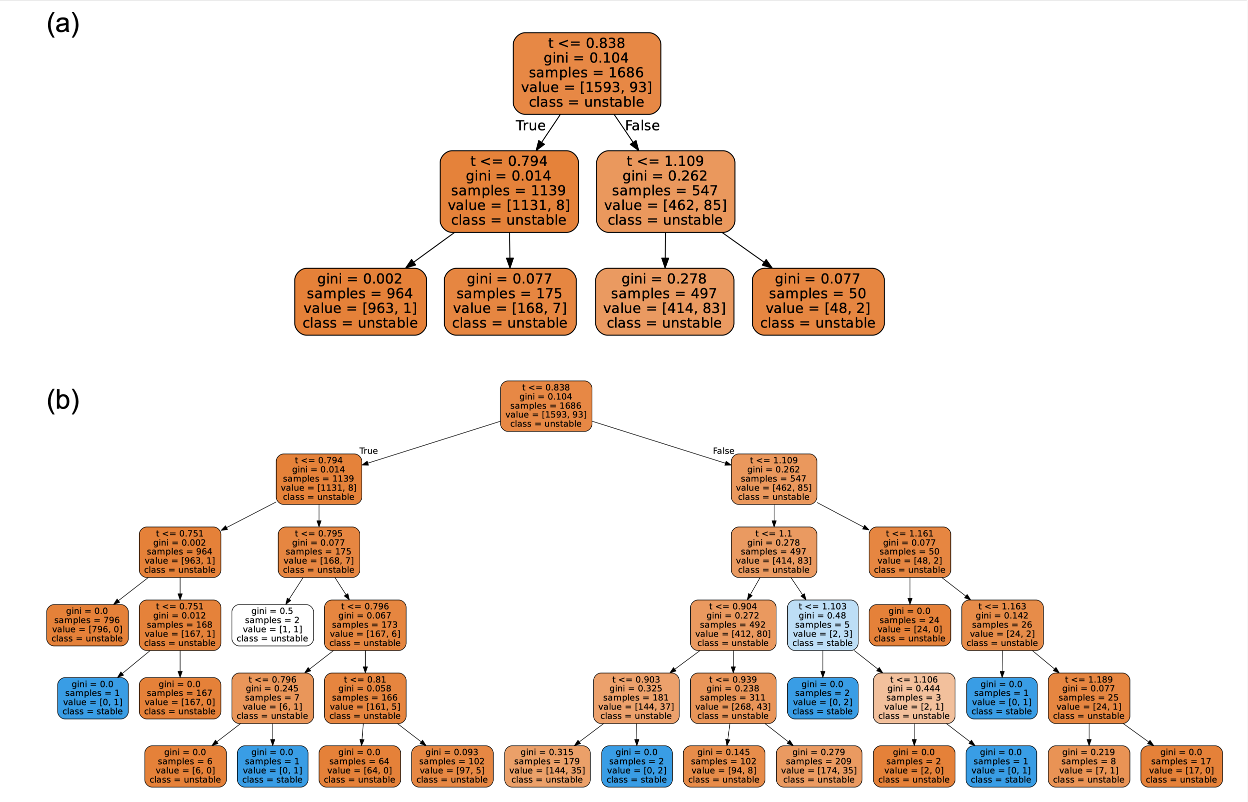

Figure S4. Analysis of the tolerance factor t_{bv} to differential meta/stable oxyhydrides using the decision tree with the max depth as (a) 2 and (b) 5, respectively. The blue boxes correspond to the stable categories, and the orange boxes refer to the unstable categories. The value in each box indicates the number of unstable (left) and stable (right) compounds, respectively.

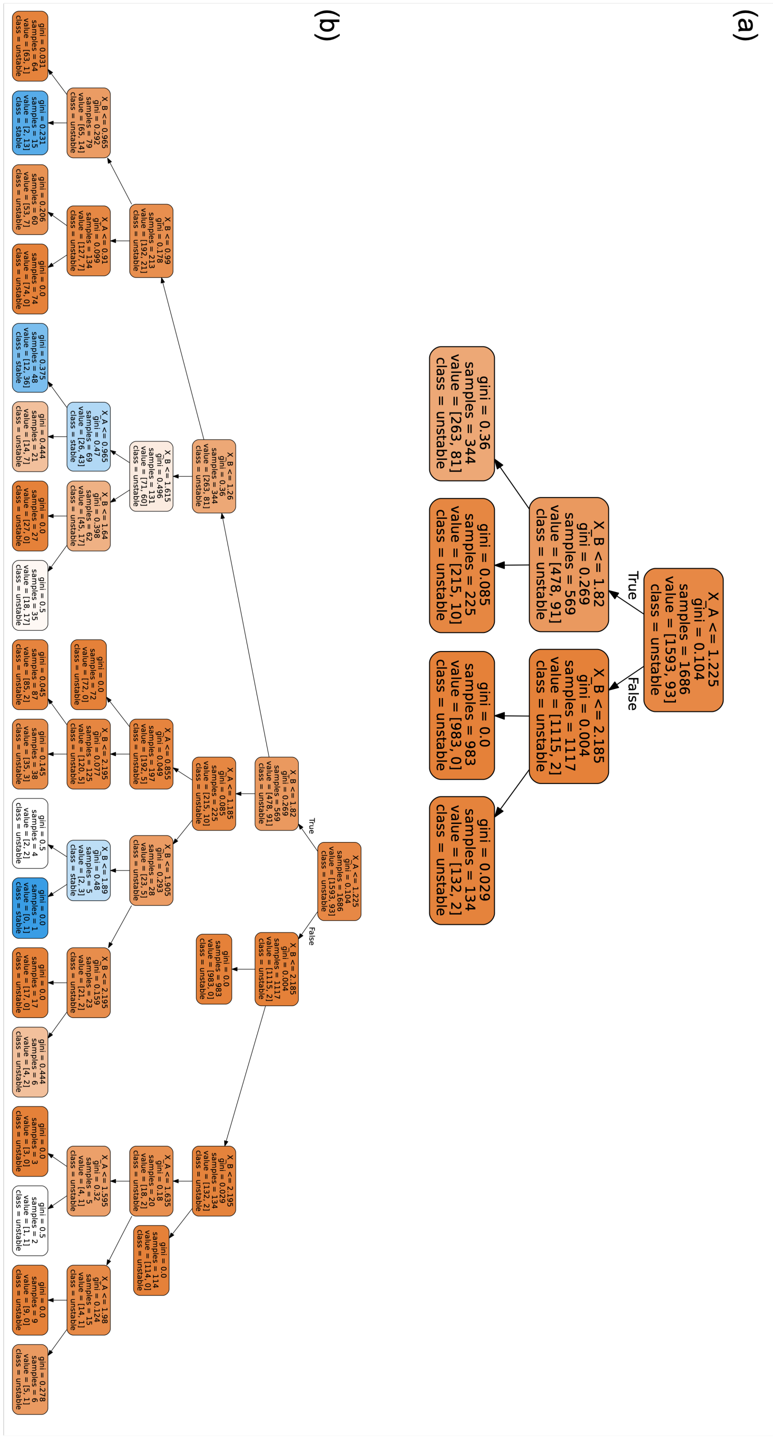

Figure S5. Analysis of the electronegativities χ_A and χ_B to differential meta/stable oxyhydrides using the decision tree with the max depth as (a) 2 and (b) 5, respectively. The blue boxes correspond to the stable categories, and the orange boxes refer to the unstable categories. The value in each box indicates the number of unstable (left) and stable (right) compounds, respectively.

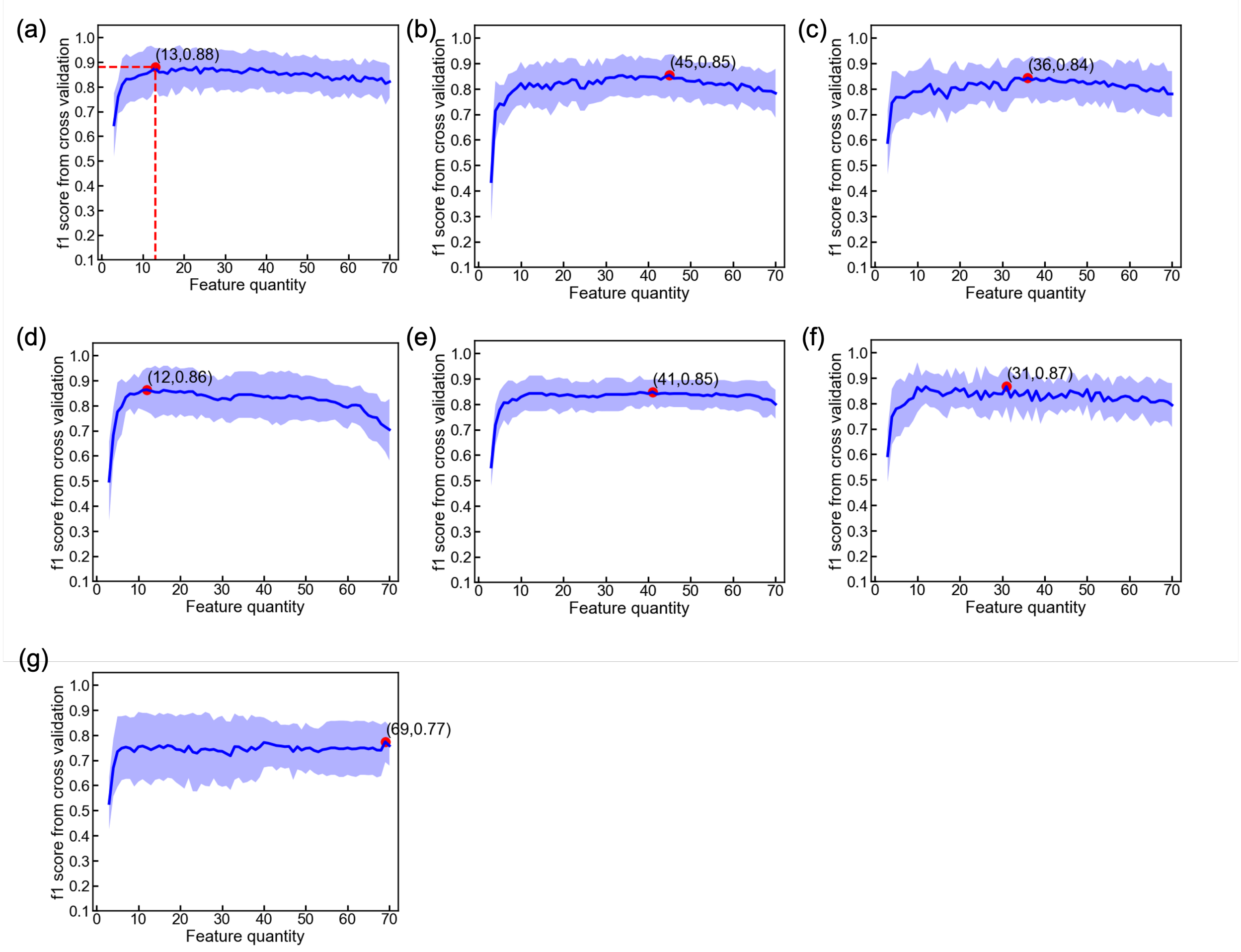

Figure S6. Ten-fold cross-validated f1 score from cross validation as a function of feature quantity using (a) voting, (b)Extra trees, (c) random forest, (d) SVC, (e) XGBoost, (f) Gradient boosting and (g) decision tree classifiers for the oxyhydrides formability using the forward sequential feature selection method. The shaded region represents the standard deviation of f1 score upon cross validation.

Table S9. The meta/stable $A_1A_2BH_2O_2$ oxyhydrides with $\Delta E_{hull} \leq 100$ meV/atom and their Bader charge of H predicted by the voting classifier. The previously known compounds are marked in bold.

Composition	ΔE_{hull} (meV/atom)	Bader charge of H (e^-)	Composition	ΔE_{hull} (meV/atom)	Bader charge of H (e^-)
$LiNaTiH_2O_2$	57	-0.67	$CsCeMgH_2O_2$	0	-0.68
$LiNaHfH_2O_2$	65	-0.71	$NaZrLiH_2O_2$	86	-0.65
$LiKTiH_2O_2$	55	-0.61	$NaCeLiH_2O_2$	89	-0.63
$LiKZrH_2O_2$	38	-0.66	$CaSrMgH_2O_2$	68	-0.77
$LiKHfH_2O_2$	33	-0.68	$CaSrMnH_2O_2$	67	-0.62
$LiRbZrH_2O_2$	57	-0.66	$CaSrZnH_2O_2$	60	-0.53
$LiRbHfH_2O_2$	57	-0.67	$CaBaMgH_2O_2$	34	-0.75
$LiCsZrH_2O_2$	94	-0.65	$CaBaMnH_2O_2$	65	-0.71
$LiCsHfH_2O_2$	97	-0.66	$CaEuMnH_2O_2$	74	-0.66
$NaKTiH_2O_2$	55	-0.64	$CaEuZnH_2O_2$	53	-0.53
$NaKZrH_2O_2$	38	-0.68	$SrBaMgH_2O_2$	34	-0.75
$NaKHfH_2O_2$	33	-0.69	$SrBaMnH_2O_2$	38	-0.62
$NaRbTiH_2O_2$	77	-0.64	$SrBaZnH_2O_2$	54	-0.54
$NaRbZrH_2O_2$	57	-0.67	$SrEuMgH_2O_2$	85	-0.79
$NaRbHfH_2O_2$	57	-0.69	$SrEuZnH_2O_2$	40	-0.54
$NaCsTiH_2O_2$	82	-0.63	$BaEuZnH_2O_2$	73	-0.52
$NaCsZrH_2O_2$	94	-0.67	$CaYLiH_2O_2$	22	-0.71
$NaCsHfH_2O_2$	97	-0.68	$CaLaLiH_2O_2$	24	-0.70
$KRbTiH_2O_2$	55	-0.63	$CaPrLiH_2O_2$	0	-0.70
$KRbZrH_2O_2$	57	-0.66	$CaNdLiH_2O_2$	14	-0.70
$KRbHfH_2O_2$	57	-0.68	$CaSmLiH_2O_2$	3	-0.70
$KCsZrH_2O_2$	94	-0.65	$CaGdLiH_2O_2$	6	-0.69
$KCsHfH_2O_2$	97	-0.67	$CaTbLiH_2O_2$	17	-0.71
$RbCsZrH_2O_2$	94	-0.65	$CaDyLiH_2O_2$	23	-0.71
$RbCsHfH_2O_2$	97	-0.66	$CaHoLiH_2O_2$	29	-0.71
$NaCaCrH_2O_2$	95	-0.53	$CaErLiH_2O_2$	35	-0.71
$NaSrCrH_2O_2$	87	-0.53	$CaTmLiH_2O_2$	43	-0.71
$NaEuCrH_2O_2$	25	-0.56	$CaLuLiH_2O_2$	57	-0.72
$KSrScH_2O_2$	68	-0.71	$SrYLiH_2O_2$	35	-0.72
$KBaScH_2O_2$	60	-0.70	**$SrLaLiH_2O_2$**	0	-0.71
$KBaCrH_2O_2$	92	-0.62	$SrCeLiH_2O_2$	61	-0.72
$KBaLuH_2O_2$	85	-0.72	**$SrPrLiH_2O_2$**	0	-0.71
$KEuScH_2O_2$	63	-0.70	**$SrNdLiH_2O_2$**	0	-0.71

$KEuCrH_2O_2$	40	-0.55	**$SrSmLiH_2O_2$**	2	-0.71
$RbSrScH_2O_2$	87	-0.70	**$SrGdLiH_2O_2$**	14	-0.70
$RbBaScH_2O_2$	73	-0.69	$SrTbLiH_2O_2$	32	-0.72
$RbBaLuH_2O_2$	92	-0.72	$SrDyLiH_2O_2$	42	-0.72
$RbEuScH_2O_2$	95	-0.69	$SrHoLiH_2O_2$	48	-0.72
$RbEuCrH_2O_2$	96	-0.55	$SrErLiH_2O_2$	60	-0.72
$CsBaScH_2O_2$	98	-0.68	$SrTmLiH_2O_2$	74	-0.72
$NaLaMnH_2O_2$	79	-0.59	$BaLaLiH_2O_2$	38	-0.70
$NaLaZnH_2O_2$	75	-0.50	$BaPrLiH_2O_2$	43	-0.71
$NaCeMnH_2O_2$	71	-0.60	$BaNdLiH_2O_2$	54	-0.71
$NaPrMnH_2O_2$	82	-0.60	$BaSmLiH_2O_2$	76	-0.71
$NaPrZnH_2O_2$	78	-0.51	$BaGdLiH_2O_2$	88	-0.70
$NaNdMnH_2O_2$	93	-0.60	$EuYLiH_2O_2$	68	-0.71
$NaNdZnH_2O_2$	89	-0.51	$EuPrLiH_2O_2$	0	-0.70
$NaSmMnH_2O_2$	99	-0.60	$EuNdLiH_2O_2$	0	-0.70
$NaSmZnH_2O_2$	93	-0.51	$EuSmLiH_2O_2$	12	-0.69
$NaEuMnH_2O_2$	24	-0.55	$Eu_2LiH_2O_2$	0	-0.68
$NaGdZnH_2O_2$	93	-0.50	$EuTbLiH_2O_2$	38	-0.71
$KLaMnH_2O_2$	82	-0.60	$EuDyLiH_2O_2$	45	-0.71
$KCeMgH_2O_2$	46	-0.76	$EuHoLiH_2O_2$	57	-0.71
$KPrMnH_2O_2$	93	-0.61	$EuErLiH_2O_2$	65	-0.71
$KEuMnH_2O_2$	46	-0.53	$EuTmLiH_2O_2$	74	-0.71
$RbCsTiH_2O_2$	83	-0.62	$LiHfLiH_2O_2$	99	-0.66
$CaEuCoH_2O_2$	56	-0.50	$SrEuCoH_2O_2$	53	-0.51

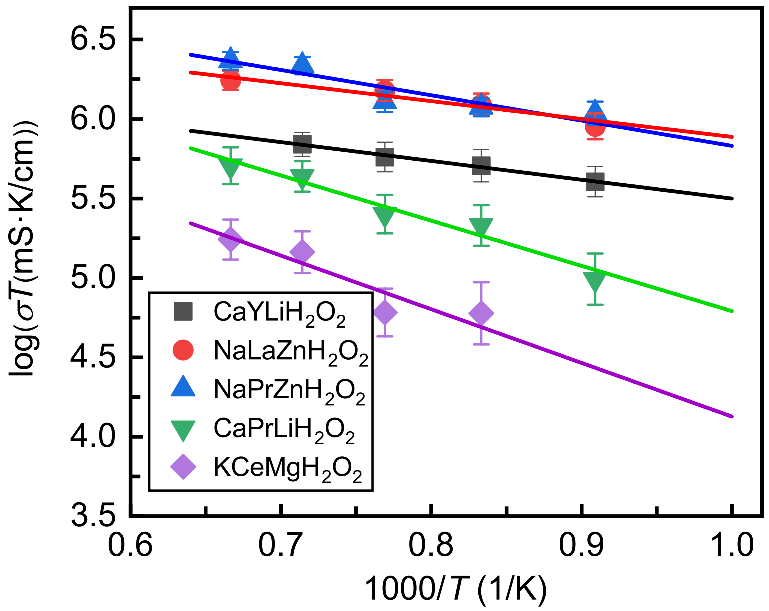

Figure S7. Arrhenius plots of $CaYLiH_2O_2$, $NaLaZnH_2O_2$, $NaPrZnH_2O_2$, $CaPrLiH_2O_2$ and L $KCeMgH_2O_2$.

Table S10. The meta/stable $A_1A_2BH_2O_2$ oxyhydrides with $\Delta E_{hull} \leq 100$ meV/atom and their Bader charge of H predicted by the random forest classifier.

Composition	ΔE_{hull} (meV/atom)	Bader charge of H (e^-)	Composition	ΔE_{hull} (meV/atom)	Bader charge of H (e^-)
$CaSmLiH_2O_2$	3	-0.70	$EuYLiH_2O_2$	68	-0.71
$EuTmH_2O_2$	74	-0.71	$SrBaZnH_2O_2$	54	-0.64
$LiKZrH_2O_2$	38	-0.66	$CaErLiH_2O_2$	35	-0.71
$NaCsHfH_2O_2$	97	-0.68	$EuTbLiH_2O_2$	38	-0.71
$SrCeLiH_2O_2$	61	-0.72	$NaSrCrH_2O_2$	87	-0.53
$RbBaScH_2O_2$	73	-0.69	$NaSrGaH_2O_2$	99	-0.53
$NaSmZnH_2O_2$	93	-0.51	$BaPrLiH_2O_2$	43	-0.71
$NaLaMnH_2O_2$	79	-0.59	$BaLaLiH_2O_2$	38	-0.70
$SrSmLiH_2O_2$	2	-0.71	$KPrMnH_2O_2$	93	-0.61
$CaBaMnH_2O_2$	65	-0.71	$SrPrLiH_2O_2$	0	-0.71
$SrDyLiH_2O_2$	42	-0.72	$KLaMnH_2O_2$	82	-0.60
$SrErLiH_2O_2$	60	-0.72	$CaTmLiH_2O_2$	43	-0.71
$NaGdZnH_2O_2$	92	-0.50	$LiCsZrH_2O_2$	94	-0.65
$RbEuScH_2O_2$	95	-0.69	$CaTbLiH_2O_2$	17	-0.71
$SrTbLiH_2O_2$	32	-0.72	$KCsHfH_2O_2$	97	-0.67
$SrLaLiH_2O_2$	0	-0.71	$SrEuMgH_2O_2$	85	-0.79
$BaNdLiH_2O_2$	54	-0.71	$SrBaMgH_2O_2$	34	-0.75
$KSrScH_2O_2$	68	-0.71	$NaPrZnH_2O_2$	78	-0.51
$KRbHfH_2O_2$	57	-0.68	$NaKZrH_2O_2$	38	-0.68
$CaSrMnH_2O_2$	67	-0.62	$CaBaMgH_2O_2$	34	-0.75
$LiKTiH_2O_2$	55	-0.61	$SrTmLiH_2O_2$	74	-0.72
$SrNdLiH_2O_2$	0	-0.71	$CaSrMgH_2O_2$	68	-0.77
$CaGdLiH_2O_2$	6	-0.69	$EuSmLiH_2O_2$	12	-0.69
$NaCeMnH_2O_2$	71	-0.60	$RbCsZrH_2O_2$	94	-0.65
$KEuMnH_2O_2$	46	-0.53	$NaLaZnH_2O_2$	75	-0.50
$NaEuCrH_2O_2$	25	-0.56	$CaDyLiH_2O_2$	23	-0.71
$NaCeLiH_2O_2$	89	-0.63	$Eu_2LiH_2O_2$	0	-0.68
$BaCsScH_2O_2$	98	-0.68	$BaEuZnH_2O_2$	73	-0.52
$BaSmLiH_2O_2$	76	-0.71	$NaNdMnH_2O_2$	93	-0.60
$SrGdLiH_2O_2$	14	-0.70	$EuNdLiH_2O_2$	0	-0.70
$NaPrMnH_2O_2$	82	-0.60	$BaGdLiH_2O_2$	88	-0.70
$CaSrZnH_2O_2$	60	-0.53	$KBaLuH_2O_2$	85	-0.72
$CaNdLiH_2O_2$	14	-0.70	$NaCsZrH_2O_2$	94	-0.67
$NaRbHfH_2O_2$	57	-0.69	$KBaCrH_2O_2$	92	-0.62
$NaCsTiH_2O_2$	82	-0.63	$RbSrScH_2O_2$	87	-0.70

$CaPrLiH_2O_2$	0	-0.70	$KCsZrH_2O_2$	94	-0.65
$KCeMgH_2O_2$	46	-0.76	$NaKHfH_2O_2$	33	-0.69
$EuErLiH_2O_2$	65	-0.71	$EuHoLiH_2O_2$	57	-0.71
$RbBaLuH_2O_2$	92	-0.72	$NaKTiH_2O_2$	55	-0.64
$KRbTiH_2O_2$	55	-0.63	$CaYLiH_2O_2$	22	-0.71
$SrHoLiH_2O_2$	48	-0.72	$NaSmMnH_2O_2$	99	-0.60
$NaCaCrH_2O_2$	95	-0.53	$NaNdZnH_2O_2$	89	-0.51
$LiRbHfH_2O_2$	57	-0.67	$KRbZrH_2O_2$	57	-0.66
$CaLaLiH_2O_2$	24	-0.70	$RbCsHfH_2O_2$	97	-0.66
$SrEuZnH_2O_2$	40	-0.54	$KEuCrH_2O_2$	40	-0.55
$SrBaMnH_2O_2$	38	-0.62	$CaHoLiH_2O_2$	29	-0.71
$LiRbZrH_2O_2$	57	-0.66	$NaRbZrH_2O_2$	57	-0.67
$LiCsHfH_2O_2$	97	-0.66	$CaLuLiH_2O_2$	57	-0.72
$EuDyLiH_2O_2$	45	-0.71	$CaEuZnH_2O_2$	53	-0.53
$KBaScH_2O_2$	60	-0.70	$LiKHfH_2O_2$	33	-0.68
$NaRbTiH_2O_2$	77	-0.64	$SrYLiH_2O_2$	35	-0.72
$CsCeMgH_2O_2$	0	-0.68	$LiNaTiH_2O_2$	57	-0.66
$NaCeFeH_2O_2$	55	-0.53	$NaEuMnH_2O_2$	92	-0.56
$NaEuSnH_2O_2$	0	-0.70	$NaTbZnH_2O_2$	98	-0.51
$NaYbMnH_2O_2$	95	-0.51			

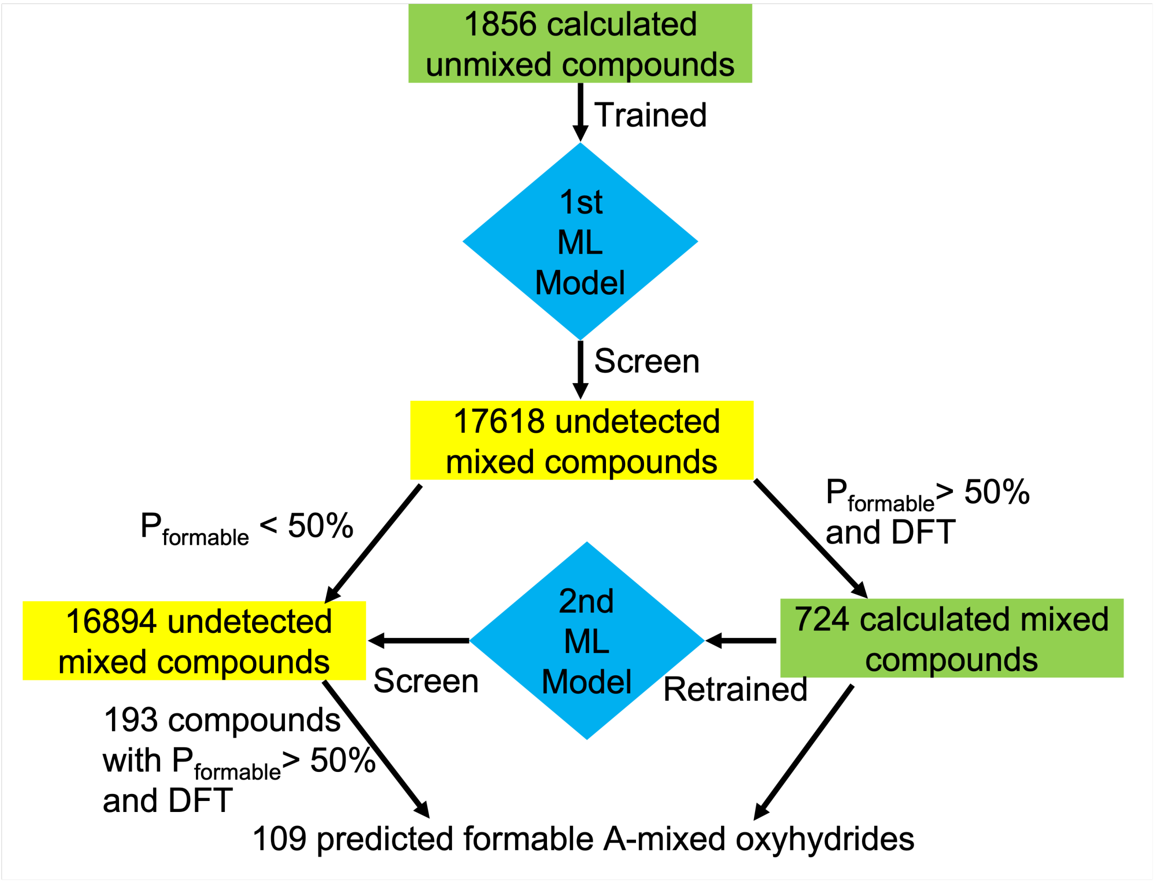

Figure S8. Workflow of the sequential machine leaning approach to search formable A-mixed oxyhydrides by the random forest model.

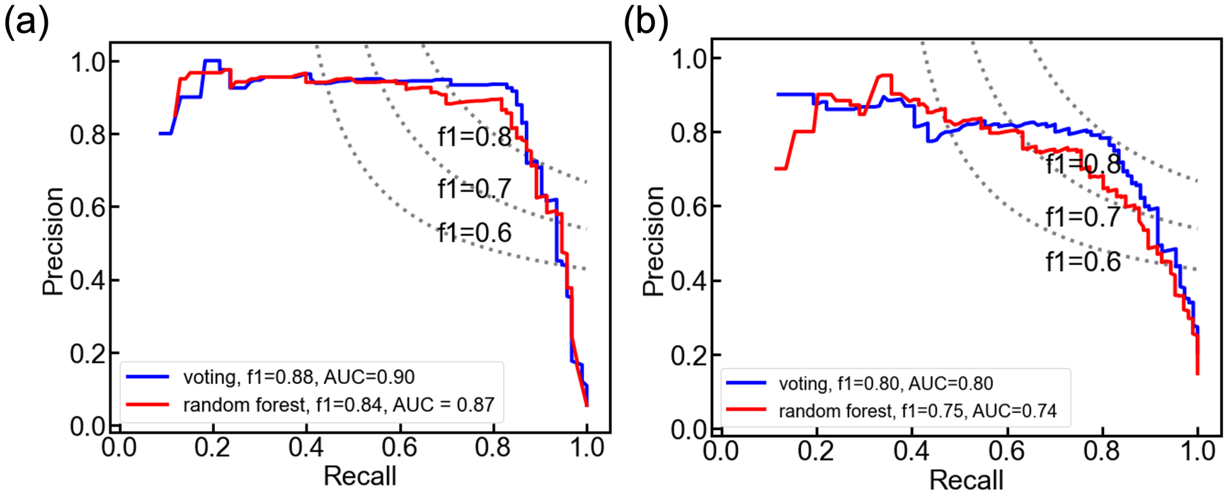

Figure S9. (a) Cross-validated precision-recall curve of the voting and random forest classifiers to identify the stable unmixed oxyhydrides (i.e., the first round ML). (b) Cross-validated precision-recall curve of the voting and random forest classifiers to identify the stable mixed oxyhydrides (i.e., the second round ML). The area under the precision-recall curve (AUC-PR) can indicate the performance of the classifier. The classifier with larger AUC-PR exhibits better performance.

Reference

1. S. Kumari, D. Kumar and M. Mittal, *International Journal of Cognitive Computing in Engineering*, 2021, **2**, 40-46.
2. F. J. Ferri, P. Pudil, M. Hatef and J. Kittler, in *Machine Intelligence and Pattern Recognition*, Elsevier, 1994, vol. 16, pp. 403-413.
3. P. Pudil, J. Novovičová and J. Kittler, *Pattern Recognition Letters*, 1994, **15**, 1119-1125.
